# Construction of a high-density genetic map for grape using next generation restriction-site associated DNA sequencing

**DOI:** 10.1186/1471-2229-12-148

**Published:** 2012-08-21

**Authors:** Nian Wang, Linchuan Fang, Haiping Xin, Lijun Wang, Shaohua Li

**Affiliations:** 1 Key Laboratory of Plant Germplasm Enhancement and Speciality Agriculture, Wuhan Botanical Garden, Chinese Academy of Sciences, Wuhan, 430074, China; 2 Graduate School of Chinese Academy of Sciences, Beijing, 100049, China; 3 Institute of Botany, Chinese Academy of Sciences, Beijing, 100093, China

**Keywords:** Grape, Genetic map, Next generation sequencing (NGS), Restriction-site associated DNA (RAD)

## Abstract

**Background:**

Genetic mapping and QTL detection are powerful methodologies in plant improvement and breeding. Construction of a high-density and high-quality genetic map would be of great benefit in the production of superior grapes to meet human demand. High throughput and low cost of the recently developed next generation sequencing (NGS) technology have resulted in its wide application in genome research. Sequencing restriction-site associated DNA (RAD) might be an efficient strategy to simplify genotyping. Combining NGS with RAD has proven to be powerful for single nucleotide polymorphism (SNP) marker development.

**Results:**

An F1 population of 100 individual plants was developed. In-silico digestion-site prediction was used to select an appropriate restriction enzyme for construction of a RAD sequencing library. Next generation RAD sequencing was applied to genotype the F1 population and its parents. Applying a cluster strategy for SNP modulation, a total of 1,814 high-quality SNP markers were developed: 1,121 of these were mapped to the female genetic map, 759 to the male map, and 1,646 to the integrated map. A comparison of the genetic maps to the published *Vitis vinifera* genome revealed both conservation and variations.

**Conclusions:**

The applicability of next generation RAD sequencing for genotyping a grape F1 population was demonstrated, leading to the successful development of a genetic map with high density and quality using our designed SNP markers. Detailed analysis revealed that this newly developed genetic map can be used for a variety of genome investigations, such as QTL detection, sequence assembly and genome comparison.

## Background

Grape (2n = 38) is one of the most important fruits worldwide, with a production of ~68 million tons over a harvested area of 7.2 million ha in 2010 (FAOSTAT, 2010). Grapes can be classified into either table or wine varieties, based on their intended mode of consumption, i.e., eaten raw or used to make wine. Consumption of grapes and wine has proven to be greatly beneficial for human health [1-4], and there has been a recent rise in the demand for high-quality grapes for human consumption. There is therefore a need to focus on grape improvement to optimize their attractive characteristics, such as contents of secondary metabolites, sugars and organic acids, resistance and yield. This can be achieved by using different germplasms from domesticated or wild-type grapes and then selecting for the genetic components that control the superior traits. However, it takes decades to produce advanced high-performing grape cultivars with the required traits, and there is still not enough resource which can produce grapes with high quality and quantity. High-density genetic map, one of the most valuable genomic resources, can largely reveal genome compositions and meet the requirement of high throughput superior traits selection among a lot of germplasms in most species, including plant and animal. Thus, construction of a high-quality genetic map for grape is necessary for its further studies and production.

In the past two decades, there have been a number of reports on the construction of grape genetic maps. Lodhi et al. [5] developed a genetic map for *Vitis* with 422 random amplified polymorphic DNA (RAPD) and 16 restriction fragment length polymorphism (RFLP) molecular markers, as well as a number of isozyme markers [5], possibly the first report of a complete genetic map for grape. From that study, a number of new genetic maps were developed, several of them based on the framework of that map. The latter studies generally made use of an F1 population as the plant material, with amplified fragment length polymorphisms (AFLP), simple sequence repeats (SSR), and single nucleotide polymorphisms (SNP) being the three major molecular marker types for map construction [6-16]. Although some genetic maps for grapes already exist, the total marker number on the linkage groups (LGs) of these existing maps is generally < 1,000 and some of these mapped markers have no sequence information. Thus a high-density genetic map for grape is still lacking, and one that covers a large number of molecular markers with sufficient sequence information is needed to meet the demand for improvement.

A key step in genetic map construction is the development of a set of testable molecular markers. In the last decade, a number of molecular marker technologies have been developed, including RAPD, AFLP, SSR and SNP. RAPD and AFLP have proven to be unstable due to many uncontrollable experimental conditions [17]. SSRs are considered to be one of the most stable and reliable markers for genetic map construction, but the experiments are time- and cost-consuming [18]. Thus, these markers are not suitable for high-density genetic map construction with high throughput. SNPs are single nucleotide polymorphisms or small InDels in the genome. They can be more numerous than other types of markers, but this is difficult to test. Before next generation sequencing (NGS) technique was developed, a number of other platforms were available for their identification, such as SNP Gene-Chip [19], high-resolution melt (HRM) analysis [20], TILLING and EcoTILLING [21,22]. With the improved sequencing technology, the last two years have seen the development of NGS combining restriction-site associated DNA (RAD) for SNP testing [23]. Pfender et al. [24] successfully used RAD markers to construct a high-density genetic map, which was subsequently employed to detect the QTL for resistance to stem rust in *Lolium perenne*. Using 2,383 RAD prior markers, an ultra-high-density genetic map was also developed for barley by Chutimanitsakun et al. [25], who showed that next generation RAD sequencing is a powerful high-throughput technique. Next generation RAD sequencing has also been successfully applied in other plants, including globe artichoke [26] and eggplant [27].

In this study, an F1 population of grape was constructed by crossing two interspecies hybridization progeny, Z180 and Beihong. Analyses of resveratrol content in the fruit skin, sugar and acid contents in the berry, berry size and cold resistance over several years revealed stable segregation of these traits in this F1 population. To take advantage of this F1 population, a high-density genetic map was constructed using next generation RAD sequencing for genotyping. The > 1,500 SNP markers contained in this map were analyzed, and aligned with the reference grapevine genome. Consequently, additional information on the genomic structures of different *Vitis* species was obtained, and the map can also be used to identify marker-linked loci that potentially control the superior traits of the two parents.

## Materials and methods

### Mapping population and DNA extraction

The F1 mapping population consisted of 100 progeny from a cross of Z180 (*V. monticola* × *V. riparia*) and Beihong (*V. vinifera* × *V. amurensis*) in 2003. Since pollen abortion occurred in Z180, Beihong was employed as the male parent. The seedlings of the two parents and their progeny were planted in the vineyard of the Germplasm Repository at the Institute of Botany of the Chinese Academy of Sciences in Beijing.

Young leaf samples (second and third leaves from the apex) were harvested from each individual F1 plant and the two parents at the beginning of the vegetative period (late spring). The samples were immediately stored in liquid nitrogen and transferred to a −70°C freezer. Young leaves (0.5 g) from each plant were ground in liquid nitrogen and their DNA extracted using the DNeasy plant mini prep kit (Qiagen). DNA concentration was measured and adjusted to the same level.

### In-silico analysis of restriction enzyme-recognition sites on the reference grape genome

The sequence of the *Vitis vinifera* Pinot noir PN40024 12x genome assembly was downloaded from the international Grape Genome Browser ( http://www.genoscope.cns.fr/externe/GenomeBrowser). Recognition sequences of 30 common restriction enzymes (data not shown) were chosen to investigate their digestion sites in the reference genome using Perl script. Total number of digestion sites, length of the resultant fragments, and their distribution were calculated from the results of the in-silico analysis.

### Sample preparation and data analysis

Sample preparation for sequencing followed that in a number of published papers for NGS combined with RAD [23-25,28], with a few modifications. Illumina Solexa adapters (2006 Illumina, Inc., all right reserved.), largely unmodified, were used for library construction. In brief, 2 μg genomic DNA from each sample (100 F1 progeny and both parents) was treated with 20 units (U) MseI (New England Biolabs [NEB]) for 60 min at 37°C in a 50 μl reaction. A quick blunting kit (NEB) was used to convert 30 μl of the digested sample to 5`-phosphorylated, blunt-ended DNA in a 50-μl reaction mixture; the reaction was performed with 30 μl of digested sample, 5 μl 10X blunting buffer, 5 μl 1 mM dNTP mix, 2 μl blunting enzyme mix and 8 μl sterile dH_2_O at room temperature for 30 min. A 3`-adenine overhang was added to the resulting samples in a 50-μl reaction with 32 μl blunt-ended DNA sample, 5 μl Klenow buffer (10X), 10 μl dATP (1 mM), 3 μl Klenow fragments (3` → 5` exo^-^, 5U/μl) and sterile dH_2_O to the final volume at 37°C for 1 h. Then 2 μl of 100 nM P1 and P2 adapter with a 3- to 5-bp plant-specific index (barcode) at the 5` end and a thymine overhang at the 3` end was added to each sample in a 50-μl reaction. The sequence of P1 and P2 adaptors: P1F: 5`-ACACTCTTTCCCTACACGACGCTCTTCCGATCTxxxT-3`(xxx indicated barcode); P1R: 5`phos-yyyAGATCGGAAGAGCGTCGTGTAGGGAAAGAGTGT-3` (yyy, reverse complement of xxx); P2F: 5`phos-AGATCGGAAGAGCGGTTCAGCAGGAATGCCGAG-3`; P2R: 5`-CTCGGCATTCCTGCTGAACCGCTCTTCCGATCTT-3`. A ligation reaction was carried out overnight at 16°C with T4 DNA ligase and 16 samples with different plant indices were pooled into one. DNA fragments from 400 to 500 bp (including the ~120-bp adaptor) were separated on a 1.5% agarose gel and purified using a MiniElute gel extraction kit (Qiagen). Finally, all pooled samples were amplified with Phusion High-Fidelity PCR Master Mix (NEB) for 18 cycles in a 100-μl reaction including 20 μl Phusion master mix, 5 μl of 10 μM modified Solexa amplification primer mix (AP1 and AP1; 2006 Illumina, Inc. , all right reserved) and sterile dH_2_O to the final volume. The AP1 and AP2 primers contained Illumina sequencing primer sites. The sequences are: AP1: 5`-AATGATACGGCGACCACCGAGATCTACACTCTTTCCCTACACGACGCTCTTCCGATCT-3`; P2: 5`-CAAGCAGAAGACGGCATACGAGATCGGTCTCGGCATTCCTGCTGAACCGCTCTTCCGATCT-3`; the underlined sequences are identical to Illumina sequencing primer sites. PCR products were repurified using the QIAquick PCR purification kit (Qiagen) and sequenced on a genome analyzer II instrument. All of these experiments were performed at Beijing's Biomarker Technologies Co. Ltd. ( http://www.biomarker.com.cn/english/).

SNP identification and F1 plant genotyping were performed according to the method of Pfender et al. [24], with a few modifications. A number of Perl scripts (Biomarker Technologies Co. Ltd.) were programmed to conduct the analysis. In brief, low-quality data were discarded (five bases with Q score < 20) first, and Solexa sequences were assigned to the 102 plants according to their given index. The first 30 bp of each read (designated as RAD tags) were employed for subsequent analysis. For SNP marker identification, a cluster analysis was performed for both parents' data together. RAD tags were compared and nearly identical tags, with one or two mismatches (SNPs or 1- to 2-bp InDels), were assigned to one cluster. Clusters with > 200 or < 5 reads were discarded. More than one mismatch on the 30-bp sequence of the same RAD tag was considered a haplotype and regarded as one potential SNP marker in the subsequent analyses. In one RAD tag cluster, mismatches among different plants in the F1 population were considered putative polymorphisms and the different mismatches were regarded as multiple alleles. The parental genotypes for each RAD tag cluster were also analyzed according to the origin of the 30-bp tag sequence. To genotype all 100 F1 plants, their 30-bp sequences were also clustered and analyzed separately following the strategy applied in the cluster analysis of the two parent RAD tags. The genotypes for each RAD tag cluster of a single F1 plant were then determined by the identity between them and the corresponding clusters in the two parents.

### Linkage map construction

Because of the lack of an anchor marker in this study, we first identified a set of SNP markers to assign the 19 grapevine chromosomes to19 LGs. This was performed in two steps: 1) we marked the segregation patterns of all 1,814 SNP markers as ab × cd, ef × eg, hk × hk, lm × ll and nn × np. Three types of markers, ab × cd, ef × eg and hk × hk, which could be mapped to both parental linkage maps, were regarded as candidate anchor markers; 2) the two representative 30-bp sequences (because all alleles of a SNP marker had two nearly identical 30-bp sequences, we could take the sequence of any allele representing the genotype of this SNP marker) of these candidate anchor markers were aligned with the sequence of the 12x genomic assembly of *V. vinifera* Pinot noir PN40024 using local BLAST software. The positions of each sequence for one SNP marker on the genome were identified by their highest number of hits. Three strict criteria were used to screen the candidate anchor marker: 1) it had to show a normal segregation ratio among the 100 F1 progeny; 2) both 30-bp end sequences had to align with the same chromosome position in the reference PN40024 genome; 3) the distance between the positions for the two end sequences on the reference genome had to fall between 200 and 500 bp (the expected size of the digested fragments was ~300–400 bp). The strategy for alignment of RAD tags with the reference genome was also used for the 1,646 SNP markers with the 19 chromosomes for subsequent comparison.

The double pseudo-test cross strategy of Grattapaglia and Sederoff [29] was applied, using JoinMap® 4.0 software, during the map construction. After data had been imported, a “CP” model was used for data mining. The ratio of marker segregation was calculated by Chi-square test. Markers showing significantly distorted segregation (*P*-value < 0.001) were excluded from the map construction. The genotypes of the 1,814 SNP markers were analyzed for linkage and recombination by applying the Kosambi function to estimate genetic map distances. To group all 1,814 markers, logarithm of odds (LOD) score thresholds ≥ 7 were used. After the LGs had been computed, their number was assigned according to the anchor markers mapped on them. The integrated map for both male and female plants was computed using the ‘Combine Group for Map Integration’ function.

## Results and discussion

### Selection of suitable restriction enzymes for RAD sequencing library construction

In this study, we did not sequence the whole genome of all F1 plants; rather, we sequenced the two ends of the ~300- to 400-bp RAD tags to simplify the grape genome and increase sequencing efficiency. Thus, selection of a suitable restriction enzyme for DNA digestion was key. Theoretically, two characteristics are required for an appropriate restriction enzyme: 1) because the NGS technology can only cover 75 to 100 bp of DNA at each end concurrently, the enzyme must be able to digest the genome of interest to a suitable size (e.g. ~300–400 bp); 2) the number of digested fragments of the expected size should be sufficient for subsequent manipulation (100,000–150,000 RAD tags). The *V. vinifera* Pinot noir PN40024 genome sequence was taken as the reference to search for an appropriate restriction enzyme.

**Figure 1:**
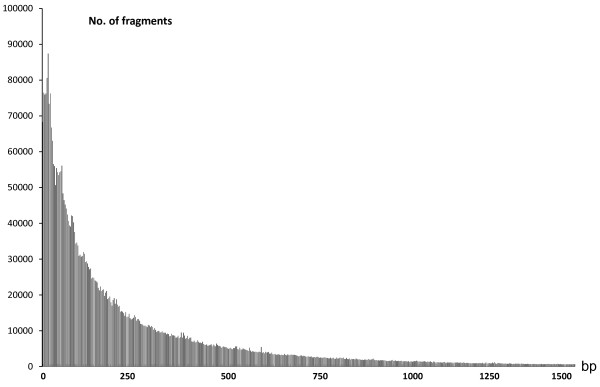
**Distribution of the restriction enzyme MseI's predicted digestion sites.** X axis indicates the size of the digested fragments; Y axis indicates the number of fragments.

In-silico digestion with ~30 restriction enzymes showed great differences in recognition sites (data not shown). One restriction enzyme, MseI, which recognized 4 nucleotides (T/TAA), was predicted to produce 149,921 digested DNA fragments for a grape genome of 300–400 bp in size, suiting our requirements. The distribution of binding sites for this restriction enzyme is shown in Figure 1. Based on these results, we selected MseI as the restriction enzyme to construct the DNA sequencing library.

### SNP markers and their characteristics

**Figure 2:**
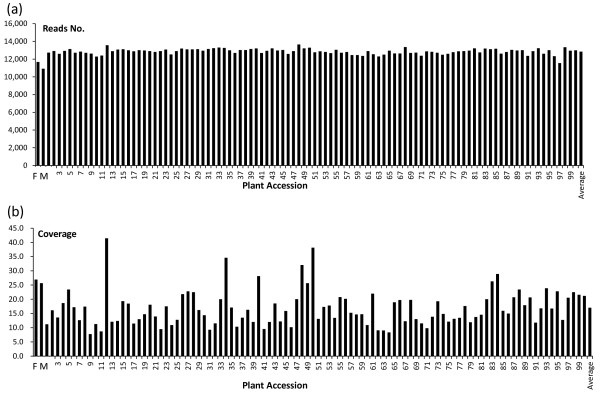
**Valid read number and coverage for each plant in the F1 population and their parents.** The X axis in **a** and **b** indicates the plant accession, including the two parents and their average one; the Y axis in **a** indicates read number, and in **b**, cluster (locus) coverage.

Once the DNA of the F1 individuals and their parents had been treated with MSeI, all samples were genotyped by high-throughput sequencing. In total, ~16 G of raw sequence data containing 117,084,991 pair-end (PE) reads was obtained, with each read being ~70 bp in length. To avoid sequence errors, only reads showing < 5 bases with Q score > 20 were further analyzed. Of these high-quality data, ~149 Mb were from one of the parents, Beihong, with 2,136,496 reads, and ~148 Mb were from Z180 with 2,126,872 reads. To assign these reads to their corresponding loci, a cluster strategy was used for the two parents' data (described in Materials and Methods). As the grape genome harbors a large number of repeat sequences [30,31], these might affect the coverage calculation and lead misidentification of polymorphisms. To overcome this obstacle, clusters containing highly redundant reads were excluded (clusters with > 200 reads), which removed the repeat sequences from the data. Clusters with a low number of reads were also excluded due to little coverage of the loci (clusters with < 5 reads). Finally, 37,871,193 high-quality reads without repeat sequences were retained, and were assigned to 80,709 clusters for the whole F1 population (Table 1). Thus we obtained 80,709 valid loci representing the whole grape genome. This number was less than the expected number of digested fragments (100,000–150,000); however, it excluded the repeat sequences and thus roughly corresponded to the in-silico digestion result. Further calculation indicated that the coverage of these loci was ~469-fold at the population level (number of valid reads: 37,871,193 per number of clusters: 80,709). With the aim of screening polymorphisms for these 80,709 clusters, a strict in-silico procedure was carried out for SNP identification (described in Materials and Methods). In total, 21,599 clusters showed more than one genotype according to their sequence diversity in the whole F1 population (Table 1). This indicated an average 26.8% polymorphism rate for the F1 population. A total of 11,144,665 reads were involved in these polymorphic loci and thus the average coverage was ~516-fold at the population level. In addition, we calculated the polymorphic loci for each F1 plant and its parents. According to Figure 2, we obtained an average of ~12,840 reads involved in the polymorphic loci and thus a 17.0-fold coverage per cluster per each individual. The reads number involved in the polymorphic loci ranged from 10,912 to 13,649 and the coverage ranged from 7.7 to 41.5-fold (Figure 2).

**Table 1:** SNP modulation for the F1 population

	**Clusters**	**No. of reads**	**Coverage**
**Polymorphisms**	21,599	11,144,665	515.98
**Non-polymorphisms**	59,110	26,726,528	452.15
**Total**	80,709	37,871,193	469.23

As already noted, the main advantages of NGS technology are low cost and high throughput. However, it also has a very serious disadvantage in its high probability of sequence error [32]. To overcome this problem, high coverage of a specific sequence must be obtained. We digested the DNA and only then sequenced the RAD tags, greatly reducing the size of the genome. Jaillon et al. ( [2007]) claimed that grapevine harbors a sequence that is ~470 Mb. During the genotyping of our 102 plants, we only manipulated ~80,709 valid clusters and each contained an ~70-bp sequence. Thus the grape genome was simplified to ~5.65 Mb (80,709 × 70 bp). This amounts to an ~83-fold reduction compared with the original 470 Mb reference genome, resulting in the requirement of very little data to achieve high coverage. According to our data, the average coverage for each tag was 17.0-fold in an individual plant. Moreover, because all sequence tags were from the two parents, Beihong and Z180, the number of alleles for each locus was ≤ 4. The total coverage for each tag at the population level was ~469-fold, leading us to adjust the SNPs in some loci where their coverage in an individual plant was insufficient. In addition, with these and subsequent strict criterions, we found the coverage of clusters corresponding to final SNP markers on the genetic map showed almost larger than 7 in an individual plant; only 24 showed from 5- to 7-fold coverage. Based on the above analyses, we concluded that the applied strategy provides high-throughput and high-quality identification of SNPs.

There were a number of possible patterns for the polymorphic markers in an F1 population (ab × cd, ef × eg, hk × hk, lm × ll, nn × np and aa × bb). However, the last pattern, aa × bb, could not be applied to the genetic map construction due to its lack of segregation in our F1 population, even though it probably constituted the largest proportion of all marker types. Thus, calculation of the segregating patterns for all loci would be necessary before a linkage map could be constructed. In addition, despite a high average coverage for the predicted RAD tag clusters, there were still a number of RAD tag clusters with low coverage in some F1 plants. To increase the accuracy of our data, only the clusters showing three or more fold coverage of > 80% of the F1 plants were used for subsequent development of SNP markers. We screened all 21,599 polymorphic clusters based on the above criteria and obtained 1,814 valid SNP markers with segregating patterns of ab × cd, ef × eg, hk × hk, lm × ll or nn × np (note that if two polymorphic clusters came from the same MseI-digested fragment, they were regarded as one marker). In addition to the coverage of the sequence data, the integrity for each locus among these 100 F1 individuals and their two parents was a key parameter in controlling map quality. We therefore investigated the data on missing rate for these plants, and found full integrity for the two parents, Z180 and Beihong, and 92.3% integrity on average for the 100 F1 plants. For a single SNP marker, the lowest integrity was ~85%, meeting the requirement for LG construction. Of these 1,814 SNP markers, 1,545 were homozygous for one parent and heterozygous for the other (960 for lm × ll and 585 for nn × np), constituting 85.2% of all selected SNP markers. However, the other three types of markers that could be mapped on both female and male linkage maps only amounted to 14.8% (ab × cd: 77, ef × eg: 171 and hk × hk: 21). This indicated that at most, 269 SNP markers could be used as shared markers for the integration of the two parents’ maps into one.

Because all of the SNP markers in this study were uniquely developed and no LG information was available, we identified a set of anchor markers that would indicate their chromosomal location. As described in Materials and Methods, the chromosome location of the 269 markers with ab × cd, ef × eg and hk × hk segregation patterns were detected according to their sequence alignment to the grape reference genome. After a series of strict selections and calculations, 212 markers clearly showed their chromosome location (Additional file 1: Table S1). Of these anchor markers, two were located on random chromosomes because the grape genomic sequence has not been completely assembled. The lowest number of anchor markers was on chromosome 15, with only two being usable for map construction (Additional file 1: Table S1). The average number of anchor markers for each chromosome was ~11.2 and only one chromosome had < 5 markers. This indicated that these anchor markers were sufficient for LG assignment.

### Genetic maps

**Figure 3:**
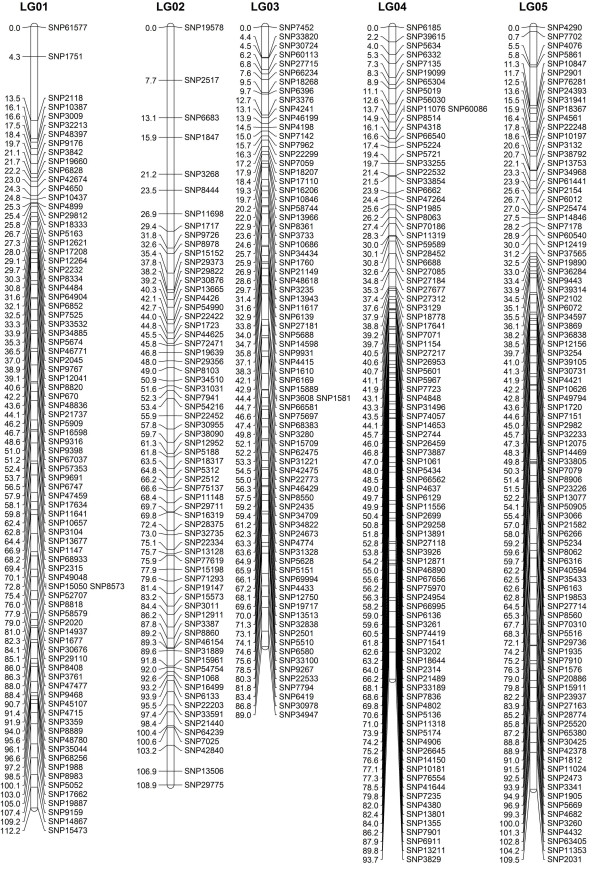
**Integrated linkage group1 to 5 for Z180×Beihong**.

**Figure 4:**
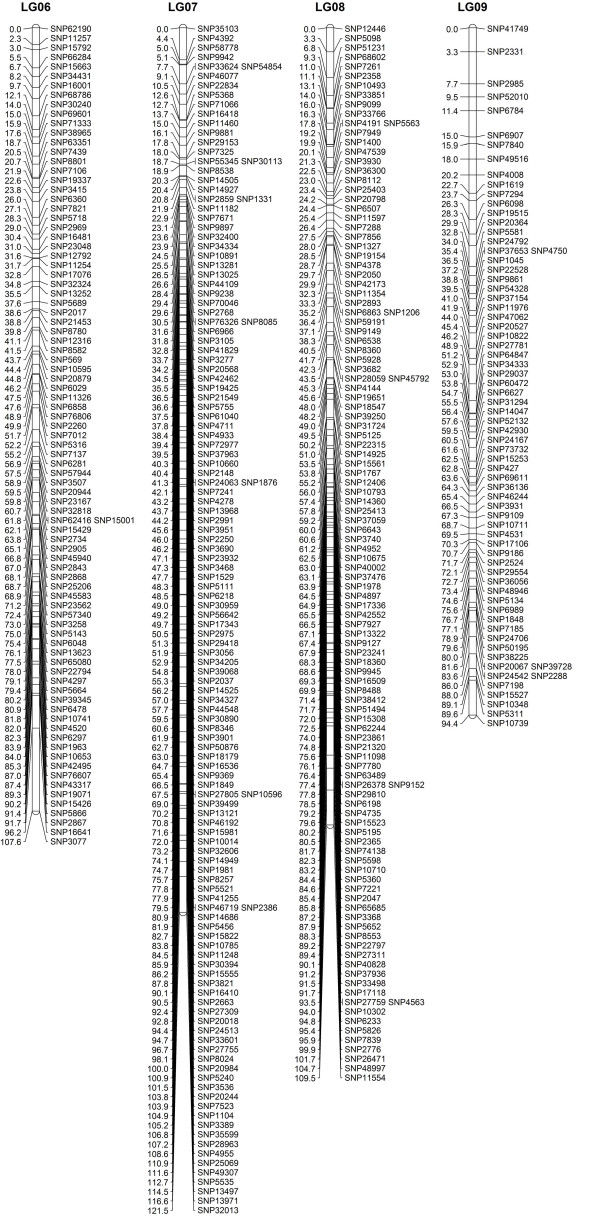
**Integrated linkage group6 to 9 for Z180×Beihong**.

**Figure 5:**
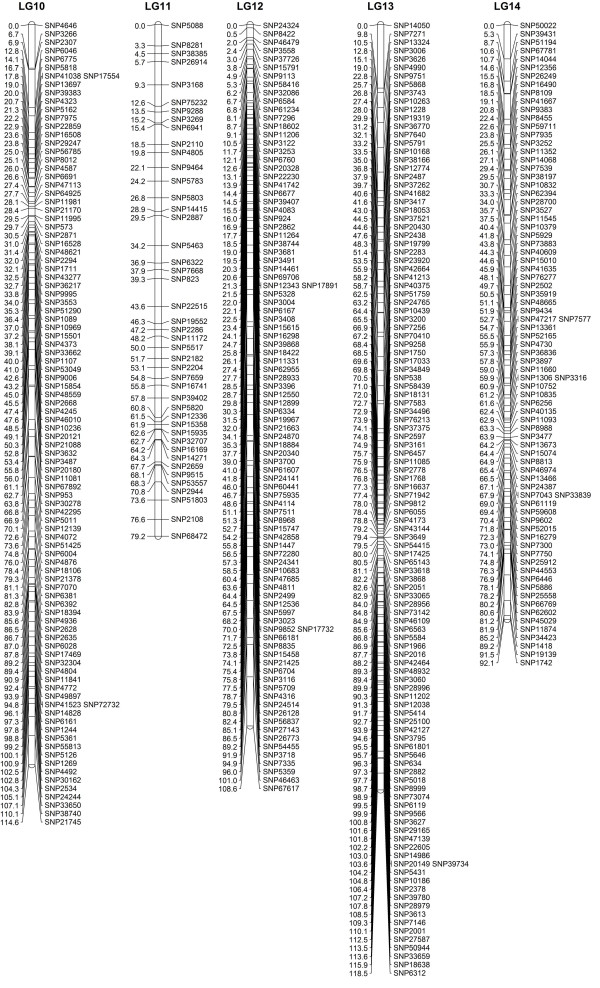
Integrated linkage group10 to 14 for Z180×Beihong.

**Figure 6:**
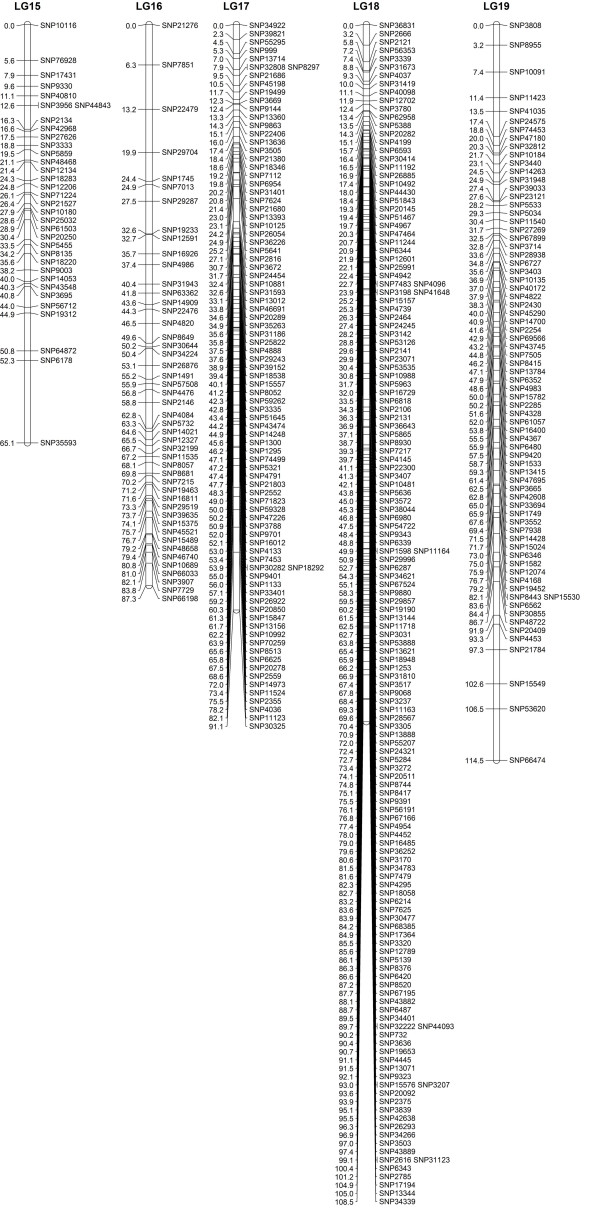
Integrated linkage group15 to 19 for Z180×Beihong.

When the data preparation was complete, the 1,814 SNP markers were imported into JoinMap4.0 for map construction. In total, 1,121 markers fell into 19 LGs for Z180 (female), 759 markers for the Beihong (male), and 1,646 markers for the integrated map, with a grouping LOD value of 7 to 13 (Figure 3, 4, 5, and 6, Additional file 2: Figure S1 and Additional file 3: Table S2). The difference in the number of markers between Z180 and Beihong might indicate the heterozygosity of Z180 is larger than Beihong; and it is corresponding to the result of an ongoing research which is conducting in our group for investigation of diversity among different vitis germplasm (unpublished). For these 19 LGs, the Z180 LG08 and Beihong LG14 did not form a uniform bar, but divided into two short LGs. Of the 212 anchor markers, 19 did not map to either Z180 or Beihong LGs, and 5 markers were specific to Beihong LGs. Thus 188 markers could be mapped on both Z180 and Beihong maps (Table S1). Further analysis of the location of the anchor markers revealed that their assignment to each chromosome by alignment to the reference genome and by LG clustering was identical. This suggested conservation of the genome structure among different species and the accuracy of our genotyping data.

Taking into account the size of all LGs, marker coverage amounted to1,884.3 cM for Z180 (female), 1,740.5 cM for Beihong (male), and 1,917.3 cM for the integrated map (Table 2). The average intervals between two adjacent mapped markers were 1.68 cM, 2.29 cM and 1.16 cM for the Z180, Beihong and integrated maps, respectively. The total physical size of the grape genome was ~470 Mb [30,31], meaning that each 1,000-kb DNA sequence was equal to an average of ~4.0 cM genetic distance in this study. Though we found there was no significant correlation between genetic and physical size in the subsequent analysis, the data still could indicate that the average intervals between two adjacent mapped markers on their genome were ~420 kb (1.68/4.0 × 1000) for Z180, ~573 kb for the Beihong, and 290 kb for the integrated map. Comparing previous reports of vitis genetic map, the total marker number on the linkage groups (LGs) of these existing maps is generally < 1,000 [6-16], therefore, the density for linkage maps developed for the F1 population of Z180 × Beihong was very high. In addition, the total sizes of grape genetic map ranged from ~1100 to ~1700 cM in previous study [6-16] and were much smaller than our map. More markers applied and interspecies crossed F1 population in this study might be attributed to this difference. More markers applied in the genetic map could detect more recombination, whereas, interspecies cross could produce more recombination. Further analysis revealed that the markers on these 19 LGs were not evenly distributed. The maximum number of markers occurred on LG18, with 95 markers for the female, 74 for the male and 148 for the integrated map. The minimum number of markers occurred on LG15—15 for Z180, 22 for Beihong and 34 for the integrated map. The size of the LGs also varied widely (Table 2): the longest LGs were LG05 for Z180 (133.2 cM), LG07 for Beihong (122.8 cM) and LG13 for the integrated map (118.5 cM); the shortest were LG15, LG11 and LG11 for Beihong, Z180 and the integrated maps, with 57.4 cM, 76.3 cM and 79.2 cM, respectively. Compared with the physical size of the corresponding chromosomes [31], the longest and shortest chromosomes were LG18 and LG17 with 34.4 and 17.9 Mb, respectively. The different physical and genetic rankings of the LGs led us to investigate the correlation between the two. Both females and males showed a very weak correlation (*r* = 0.25) between genetic and physical size among these 19 LGs/chromosomes, which might indicate that different recombination rates exist on the different chromosomes during meiosis.

**Table 2:** Genetic map for 19 linkage groups (LGs)

	**Number of markers**			**Genetic sizes (cM)**			**Chromosome size (Mb)**^**a**^
	**Female (Z180)**	**Male (Beihong)**	**Integrated map**	**Female (Z180)**	**Male (Beihong)**	**Integrated map**	
**LG01**	63	30	85	118.0	80.1	112.2	23.6
**LG02**	46	31	68	101.1	79.5	108.9	18.7
**LG03**	55	40	78	80.0	79.0	89.0	20.5
**LG04**	71	35	94	94.1	83.7	93.7	23.9
**LG05**	71	32	93	133.2	100.5	108.8	25.4
**LG06**	62	38	88	90.4	93.9	107.6	21.5
**LG07**	87	58	133	113.7	122.8	116.5	22.4
**LG08**	79	53	117	103.3	92.6	109.5	22.4
**LG09**	51	30	70	85.6	100.1	94.4	23
**LG10**	66	42	98	108.9	112.4	114.6	18.8
**LG11**	28	23	44	92.2	76.3	79.2	20.0
**LG12**	64	49	94	103.3	97.5	108.1	24.2
**LG13**	83	53	115	120.4	91.1	118.5	27.4
**LG14**	53	39	80	107.5	81.5	92.1	30.3
**LG15**	15	22	34	57.4	85.5	65.1	20.3
**LG16**	28	27	47	83.1	87.2	87.3	22.8
**LG17**	50	53	87	91.8	76.9	88.8	17.9
**LG18**	95	74	148	104.8	111.8	108.5	34.4
**LG19**	54	30	73	95.5	88.3	114.5	24.02
**Total**	1121	759	1646	1884.3	1740.5	1917.3	441.6

^a^ the physical sizes are according to Jaillon et al. ( [2007]).

A number of future studies can be based on the high-density genetic map developed in this work. First, several excellent traits exist in one of the two parents. Thus, a given trait might be improved by selection of markers which are linked to elite loci or alleles after QTL detection. Moreover, several excellent traits might be combined in one grape plant, thereby producing a new cultivar, through a series of crosses and marker-assisted selection (MAS). Second, compared to other genetic maps for grape, there are two obvious advantages: high density and complete sequence information for all markers (Additional file 3: Table S2). These advantages could greatly benefit comparative mapping and genome assembly. The markers' combined 60-bp sequences mapped to the LGs could be used as anchors for the genome. Although the genome sequence of grapevine was published several years ago, it still has a number of gaps and random sequences [30,31]. In this study, a set of markers could be aligned to the random chromosomes of *V. vinifera* Pinot noir PN40024 (data not shown). According to their positions on LGs, it might be easy to put the random chromosomes into the common one. On the other hand, the published grape genome is only for *V. vinifera,* and the genome structures of different *Vitis* species are expected to be more or less different due to the long evolutionary history of the Vitaceae [33]. Thus, comparing the genome characteristics of the different species could give us a better understanding of grape. The 1,646 mapped markers' combined 60-bp sequences could be used as shared anchors to compare genetic and physical maps (Additional file 3: Table S2). These studies might facilitate use of the grape genomic resource.

### Comparison of genetic and physical maps

To compare the genetic and physical maps, we investigated the locations of all 1,814 SNP markers on the reference genome. The high-quality 30-bp sequences from both ends of each SNP marker were employed for the location search by aligning them to the reference genome. A total of 1,456 SNP markers showed a match between their two ends and the same positions (intervals of 200–500 bp) on the reference genome; 106 markers only showed a match for one end to one position on the reference genome, while the other end had no match; the remaining 252 markers showed no match to the reference genome, showed a conflict in matching positions for the two ends, or were mapped on the random genome. To increase accuracy, only the first type of markers (1,456 SNP markers) was used to compare the genetic and physical maps.

**Figure 7:**
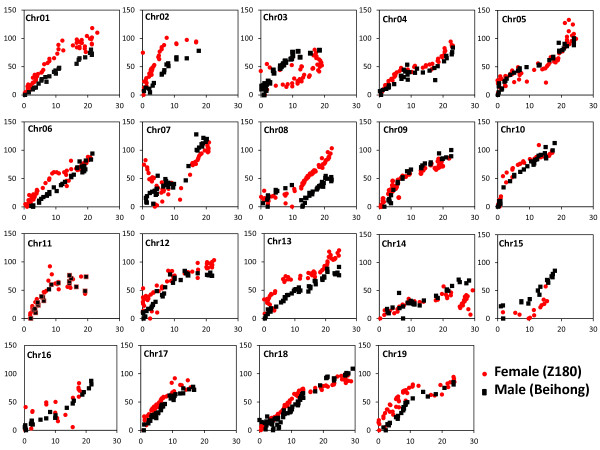
**Collinear analysis of the consensus between genetic and physical maps.** The X axis indicates the physical position of each SNP marker; the Y axis indicates the genetic position of each SNP marker. Red diamonds indicate the female genetic position against the reference physical position; black diamonds indicate the male genetic position.

From Tables 3 and Additional file 3: Table S2, 892 common markers were found between the physical and Z180 (female) genetic map; 606 common markers were found between the physical and Beihong (male) genetic map. This indicated that 79.6% (892/1,121) of the markers on the female LGs could be mapped on the reference genome; similarly, 79.8% of the markers on the male LGs could be mapped on the reference genome. Among the 19 chromosomes or LGs, LG18 showed the highest number of common markers between the physical and genetic maps for Z180 and Beihong (75 and 61, respectively); LG15 showed the lowest number of common markers, only 13 for the Z180 map and 15 for the Beihong map. To compare the order of the common markers, a dot-plot diagram (Figure 7) was generated using the physical position of each common marker on the reference genome against its genetic position on the LGs; at the same time, all LGs of the two parental maps were aligned with the reference genome (Additional file 4: Figure S2). According to these two analyses, most of the markers showed good linear agreement between physical and genetic maps on the basic framework. However, there were also chromosomes showing rearrangement of some regions. Among the 19 LGs, Chr01, 03, 04, 05, 06, 08 (two LGs for male), 09, 10, 12, 13, 14, 17, 18, 19 showed high collinear results for both female and male maps. The remaining LGs only showed high collinear results for one map. Because both parents were produced by interspecies crosses (*V. monticola* × *V. riparia* and *V. vinifera* × *V. amurensis*), some of the regions in the two parent genetic maps might be identical to the reference genome (*V. vinifera*); nevertheless, most of the regions are expected to come from the other three *Vitis* species. Therefore, the same order for the two types of map most probably indicates conservation of genomes among the different grape species; the non-collinearity for some chromosome regions might indicate some variations among different grape species during evolution.

**Table 3:** Number of common markers between genetic and physical maps for 19 individual chromosomes

**Marker names**	**Number of markers**	
	**Z180**	**Beihong**
**LG01**	55	24
**LG02**	36	18
**LG03**	50	36
**LG04**	50	31
**LG05**	64	30
**LG06**	53	32
**LG07**	54	40
**LG08**	68	41
**LG09**	43	27
**LG10**	35	27
**LG11**	25	20
**LG12**	50	37
**LG13**	62	45
**LG14**	45	31
**LG15**	13	17
**LG16**	21	22
**LG17**	47	42
**LG18**	75	61
**LG19**	46	25
**Total**	892	606

Moreover, using the high-quality, high-density genetic map, we could investigate not only the variation in genome structure among different species but also the variation that occurred during interspecies crosses. There have been a number of studies on interspecies hybridization in the past decade. Chromosome rearrangement, retrotransposon activation and SSR mutations have been seen in interspecies crosses between different types of *Brassica* species [34]. For our plant material, the parents came from two separate interspecies crosses (*V. monticola* × *V. riparia* and *V. vinifera* × *V. amurensis*), and thus the population contained four grape pedigrees. An overview comparative analysis of the genetic map and the reference genome (Figure 7) reveals a number of markers in some regions that were not in the same order. Moreover, we achieved a similar result by comparing the Z180, Beihong and integrated genetic maps (Additional file 2: Figure S1). The variations among the different species might be the first reason for this non-uniformity; however, genomic variation occurring due to *Vitis* interspecies crosses might also exist because we observed variations in the positions of a number of markers as a uniform block between the male genetic map and the *V. vinifera* physical map; the male parent (Beihong) harboured half of the *V. vinifera* pedigree. Thus, with our detailed and complete investigation of the genetic map, more knowledge of the variation among different species and interspecies crosses can be obtained in the future.

## Conclusions

We constructed a genetic map of a Z180 × Beihong F1 population of high density and quality. According to the analysis of the SNPs and their sequence information, we conclude that next generation RAD sequencing is a powerful strategy for genotyping. With further characterization of the genetic map, variations and conservation between the genetic map and reference genome were clearly detected. This genetic map is expected to be useful for QTL detection, sequence assembly and genome structure comparisons.

## Authors' contributions

NW and SL organized the entire project. LF, HX and LW harvested the leaf samples and extracted DNA for all plants. NW performed the genotyping, SNP identification, and genetic map construction. NW also wrote this manuscript, and SL and NW edited it. All authors read and approved the final manuscript.

## Supplementary Material

Additional files 1 **Table S1.** Information on anchor markers. Click here for file

Additional files 2 **Figure S1.** Genetic maps for Z180 (female), Beihong (male) and their integration. Click here for file

Additional files 3 **Table S2.** SNP markers on the 19 linkage groups (LGs) and their presented sequence. Click here for file

Additional files 4 **Figure S2.** Comparison between Z180 (female), and Beihong (male) genetic and physical maps. The SNP markers in blue are common markers between one of the two parents and the physical maps. Click here for file
