# Supplementary figures and images for: Construction of a high-density genetic map for grape using next generation restriction-site associated DNA sequencing
Source: BMC Plant Biol. 2012 Aug 21;12:148. doi: 10.1186/1471-2229-12-148 (PMC3528476; doi:10.1186/1471-2229-12-148)

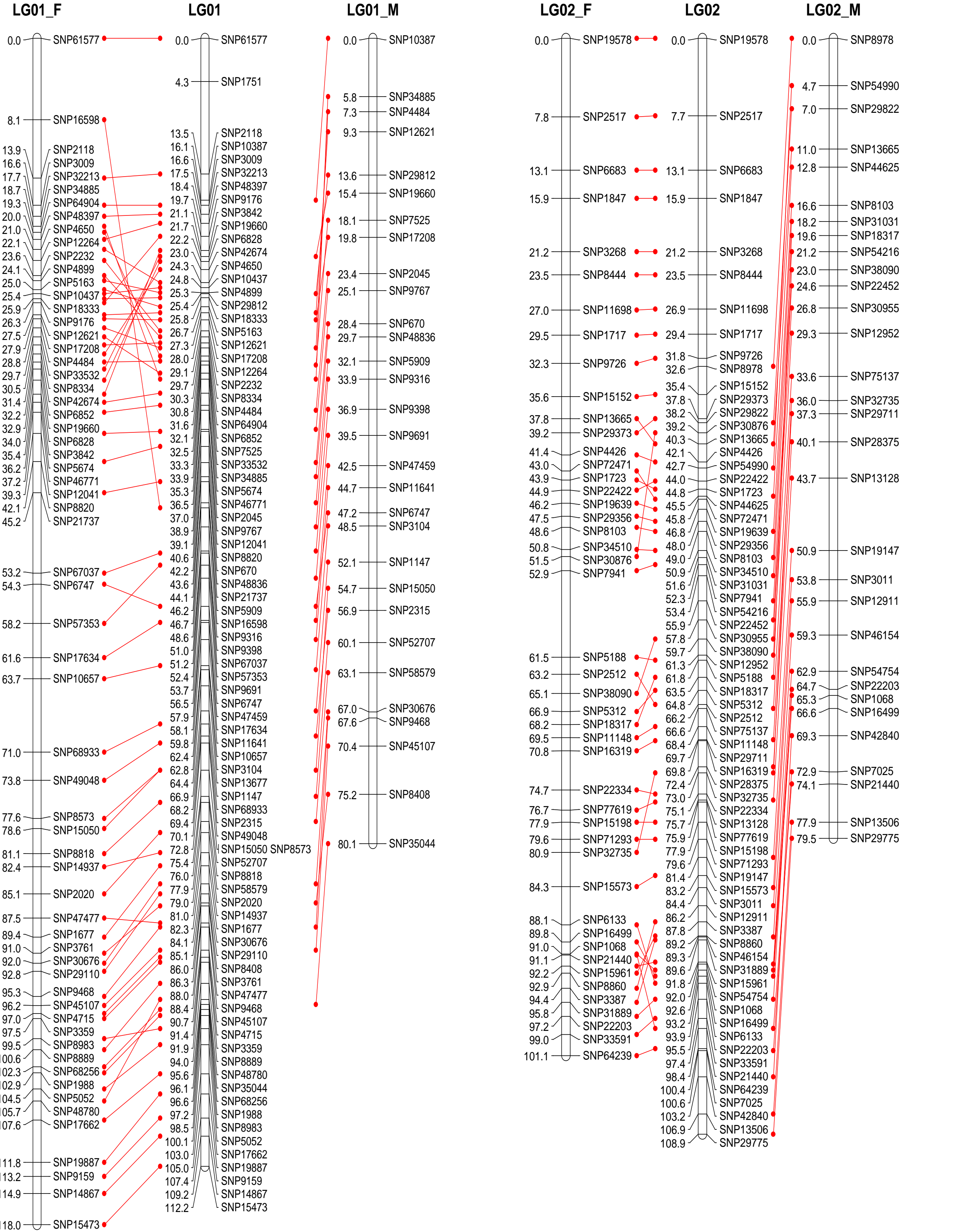

LG03\_F

LG03

LG03\_M

LG04\_F

LG04

LG04\_M

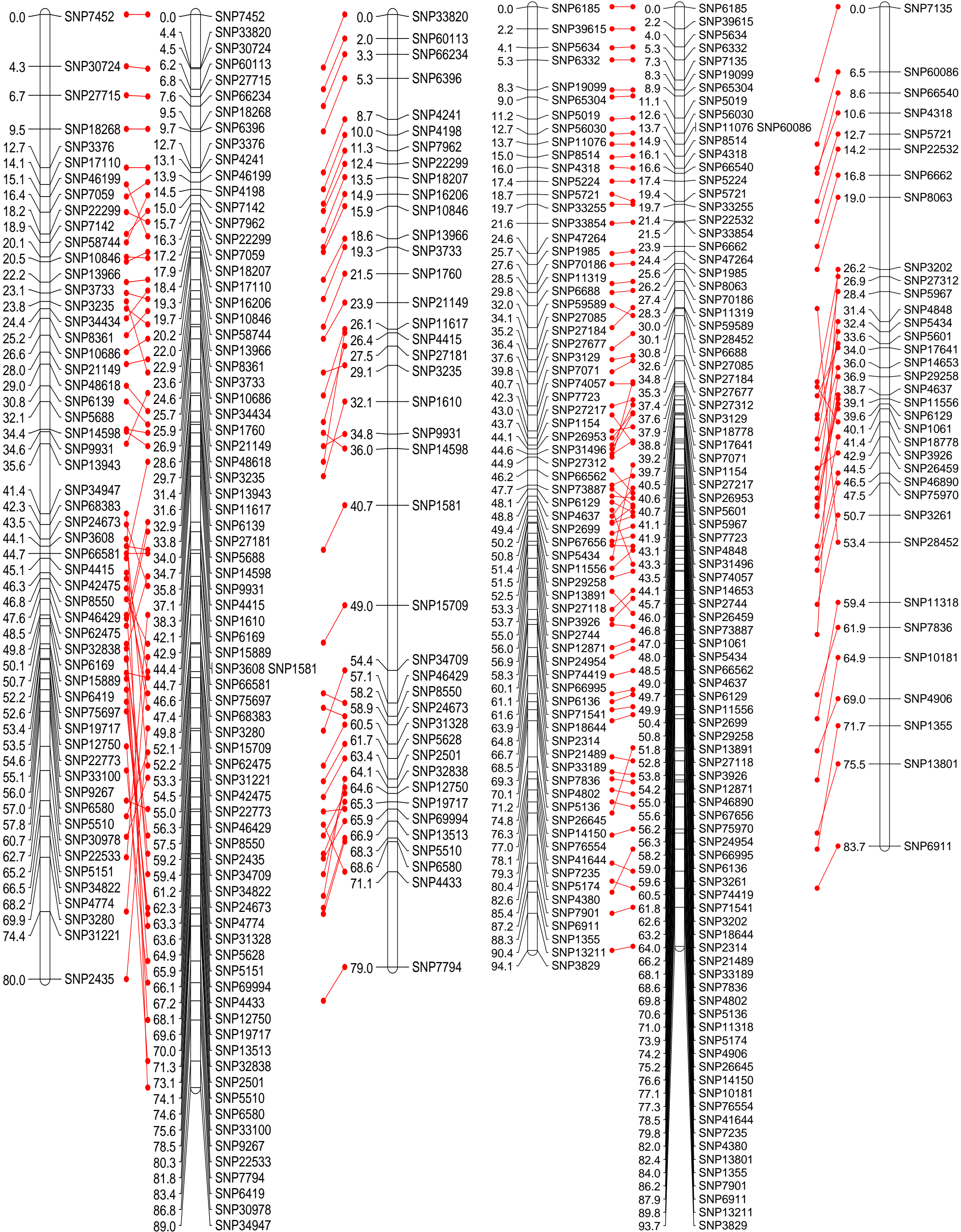

LG05\_F

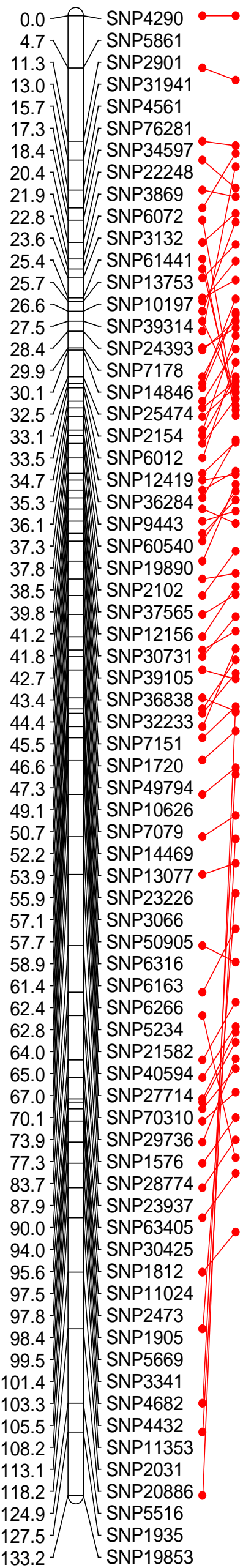

LG05

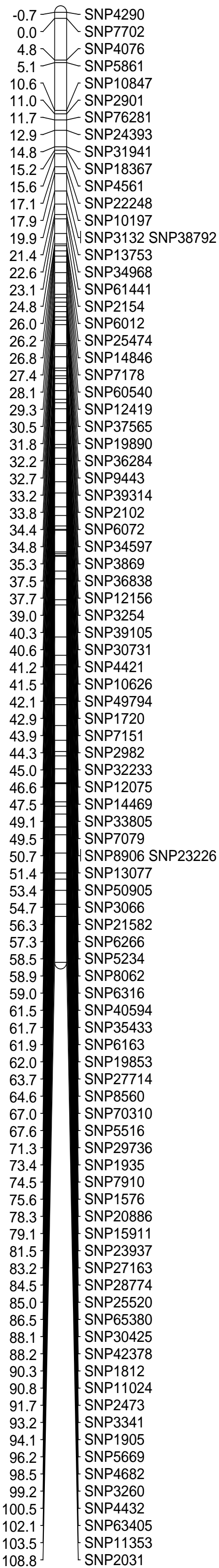

LG05\_M

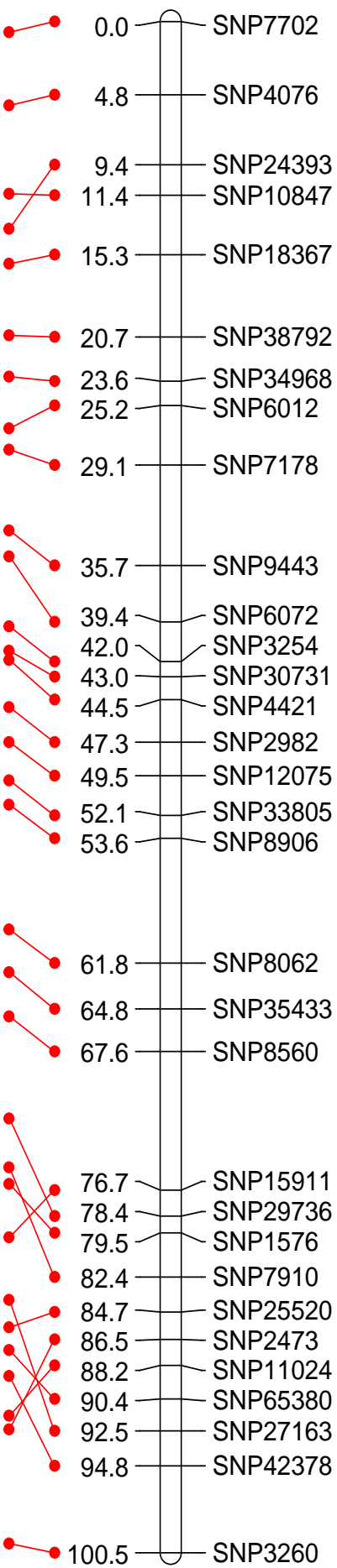

LG06\_F

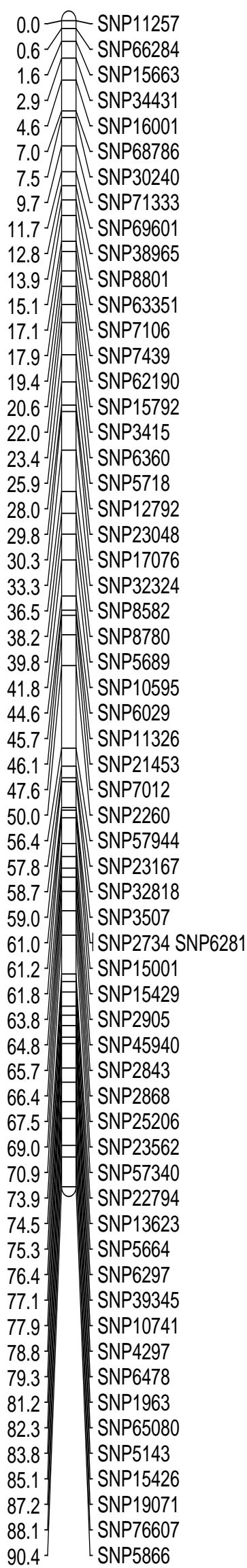

LG06

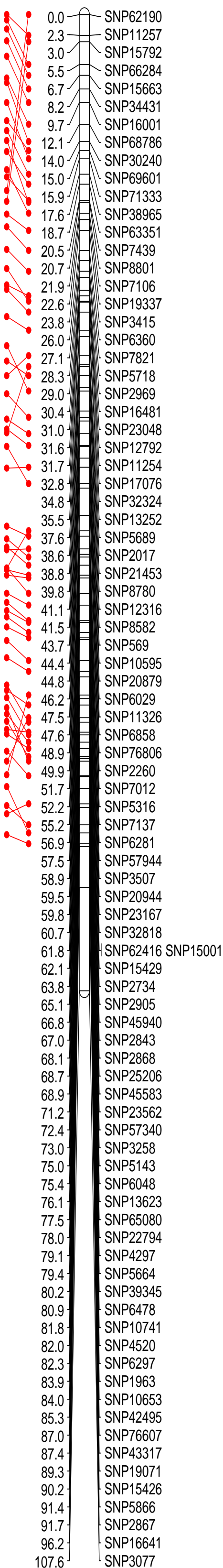

LG06\_M

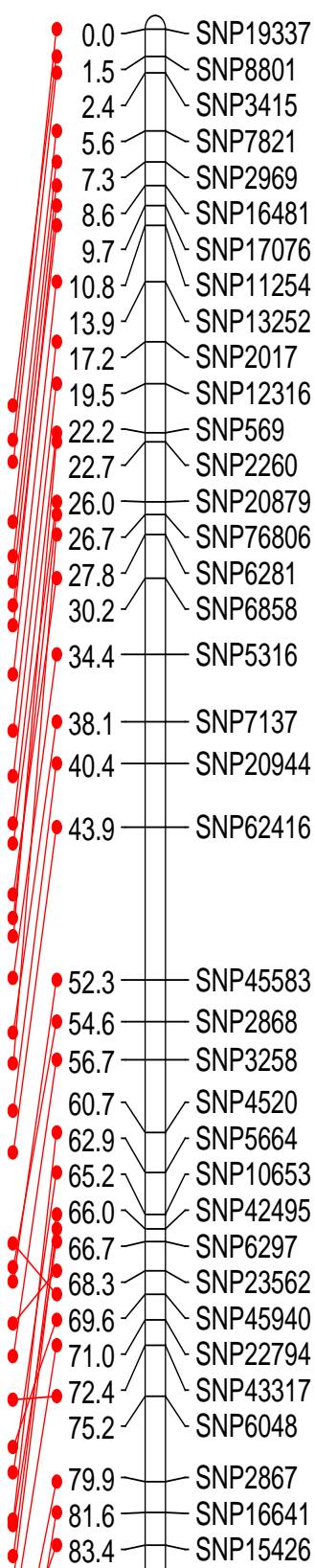

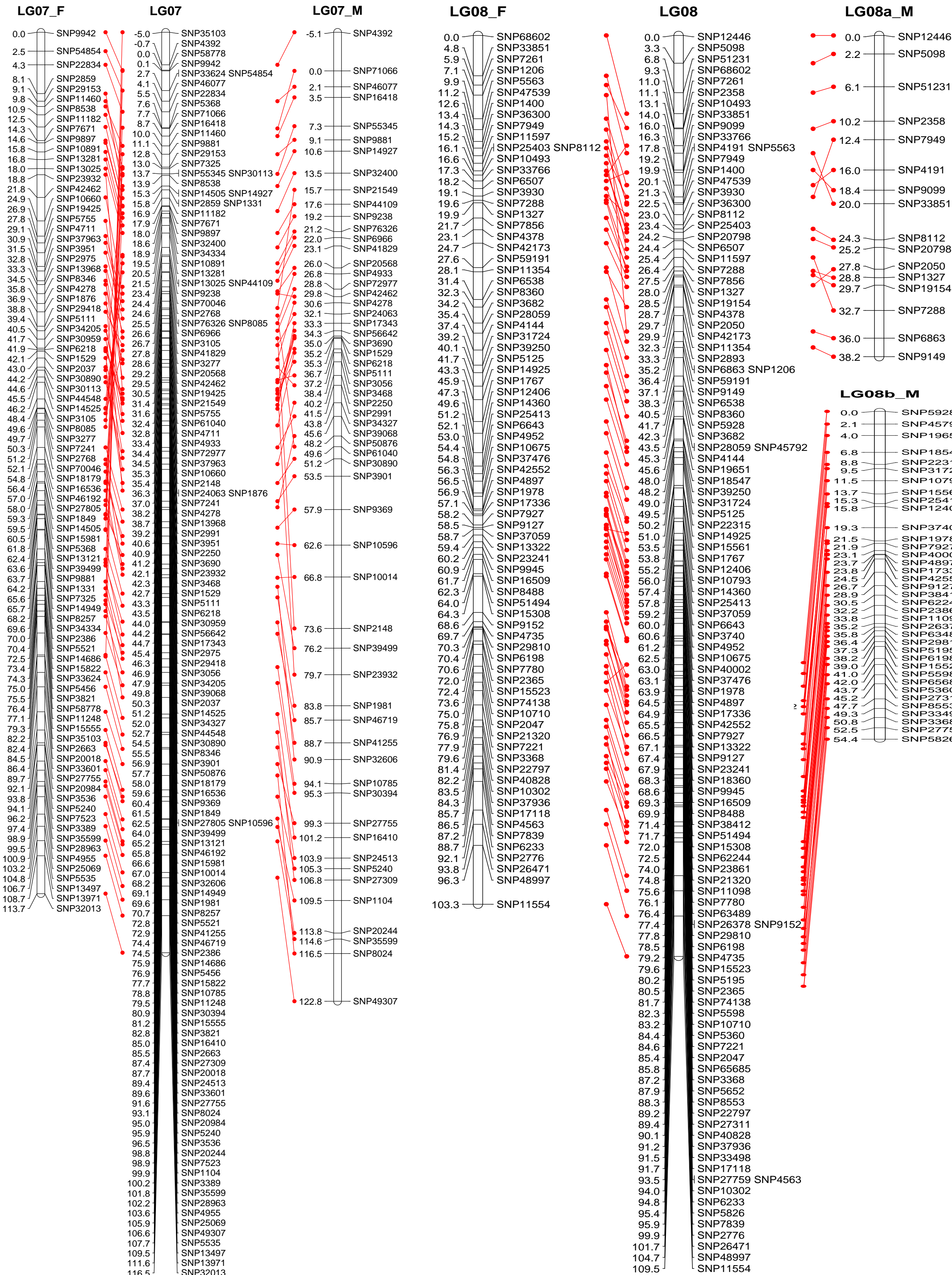

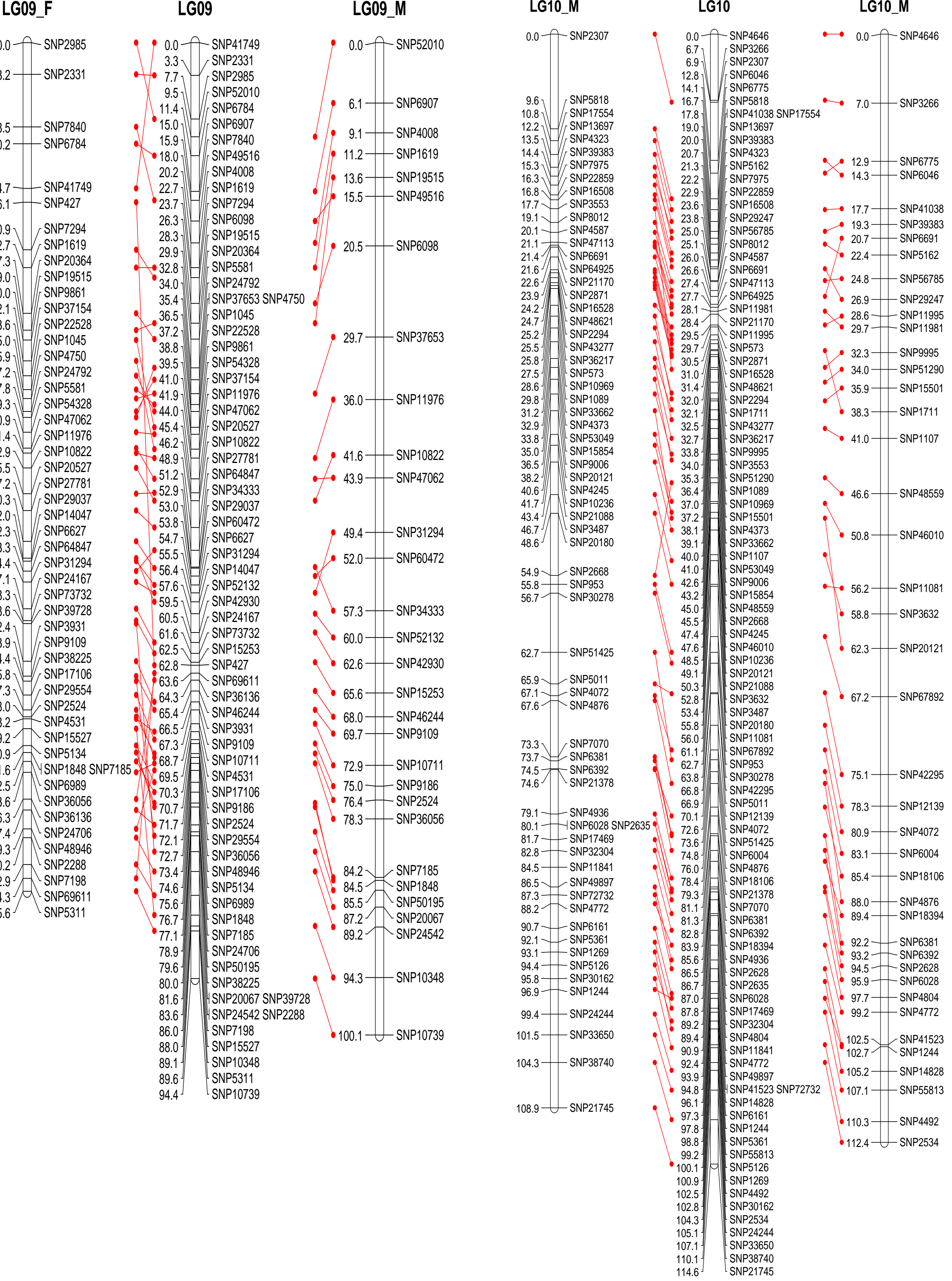

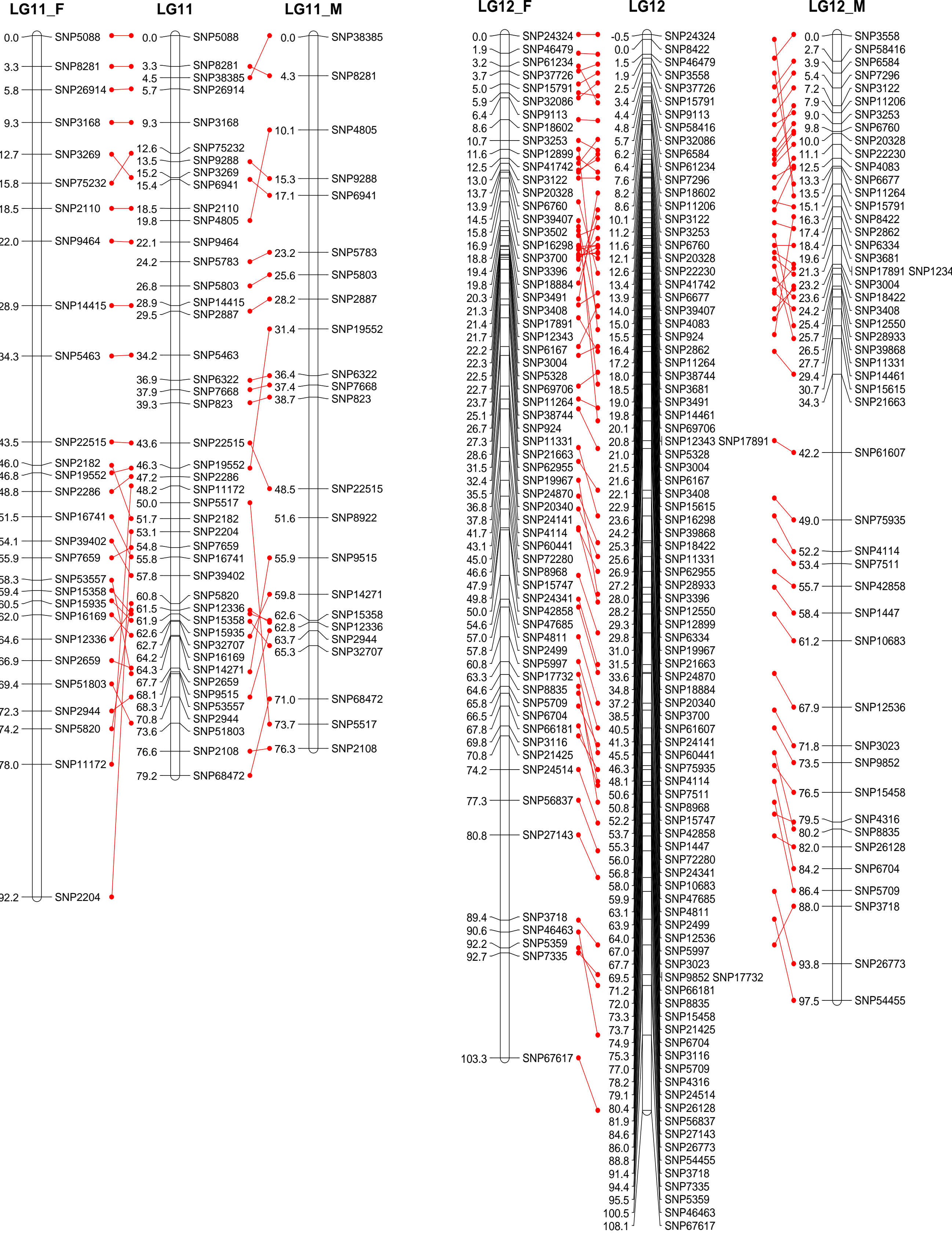

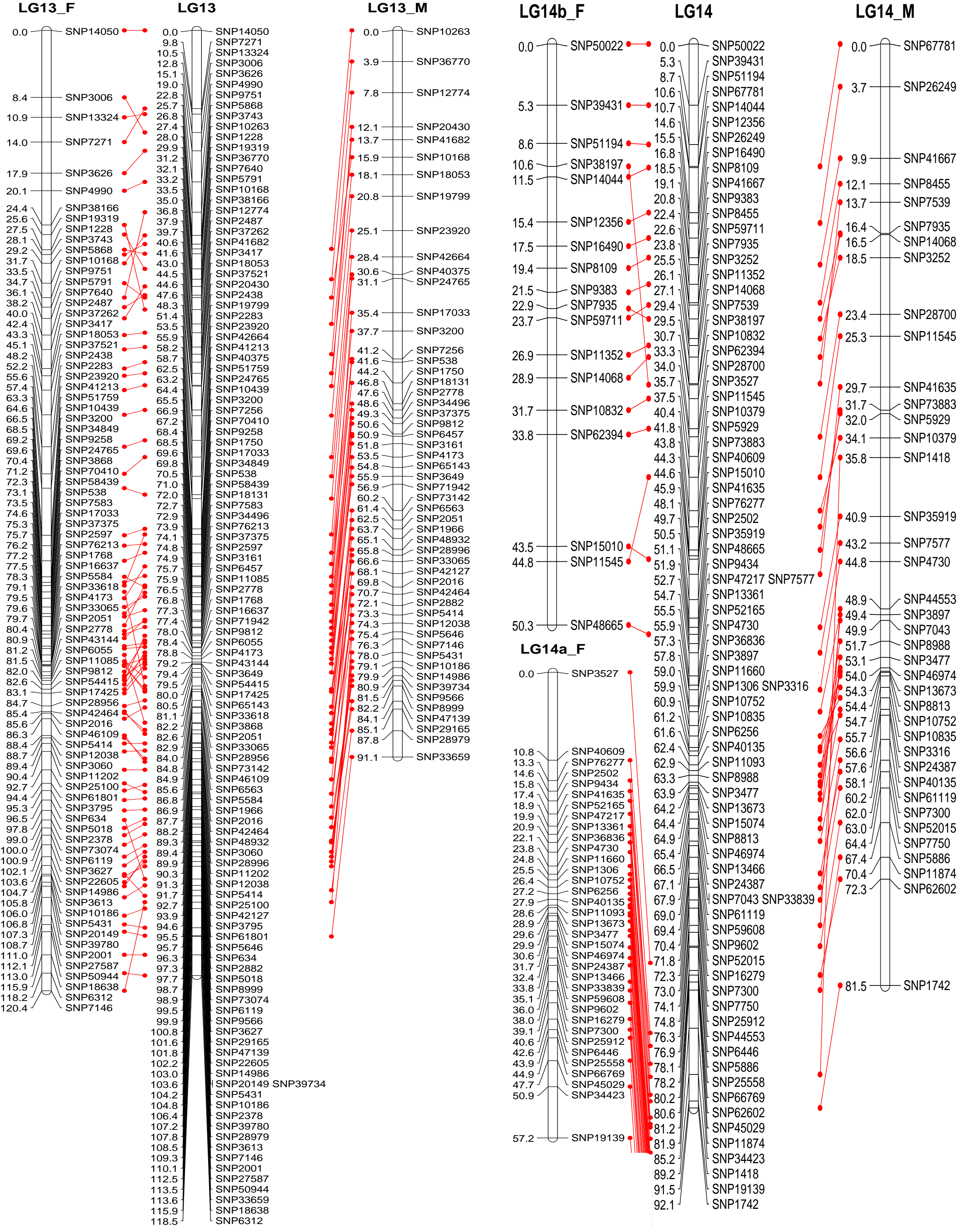

LG15\_M

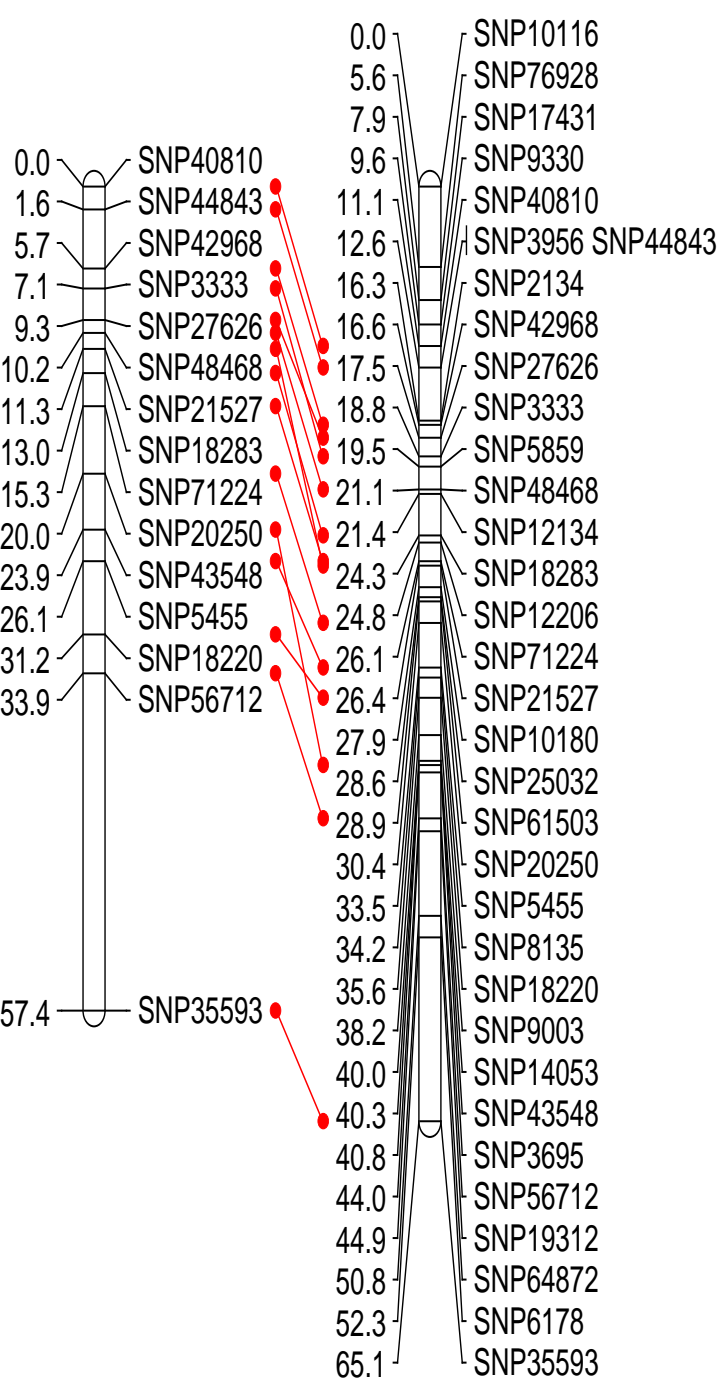

LG15

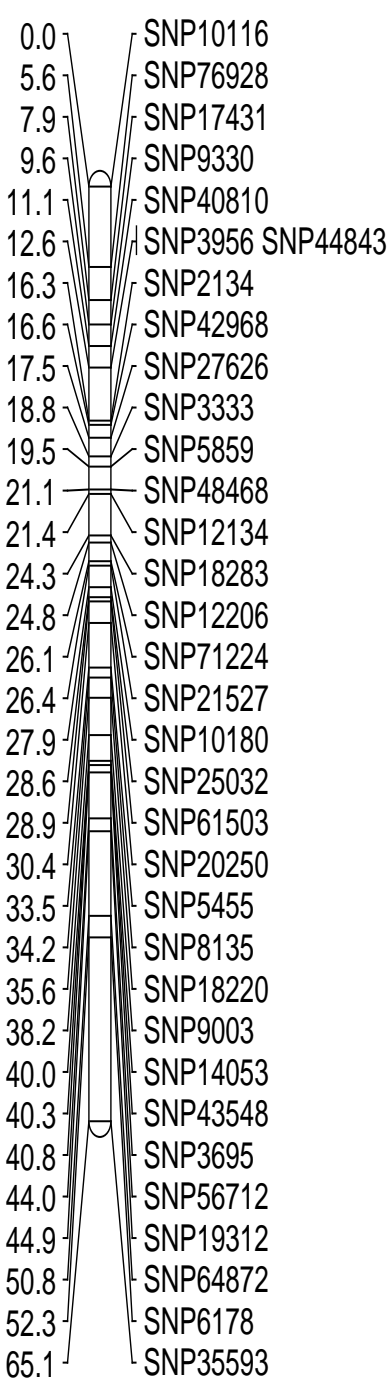

LG15\_M

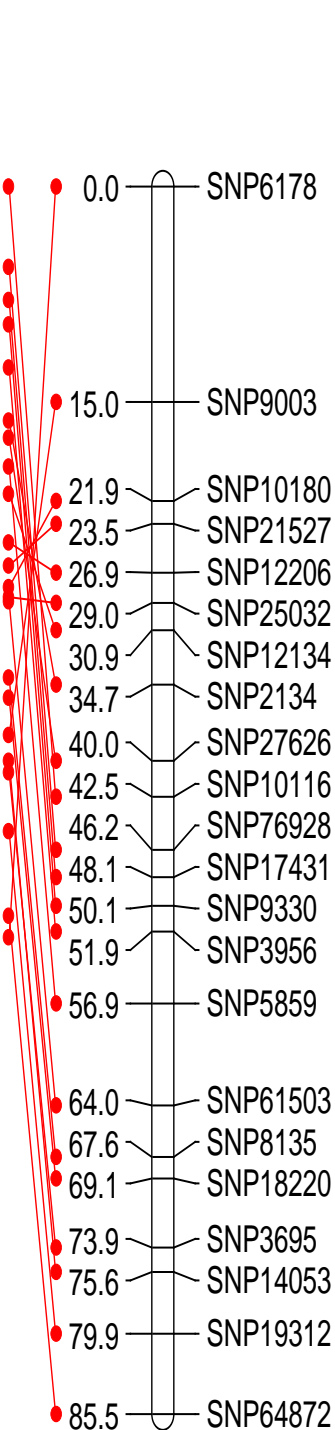

LG17\_F

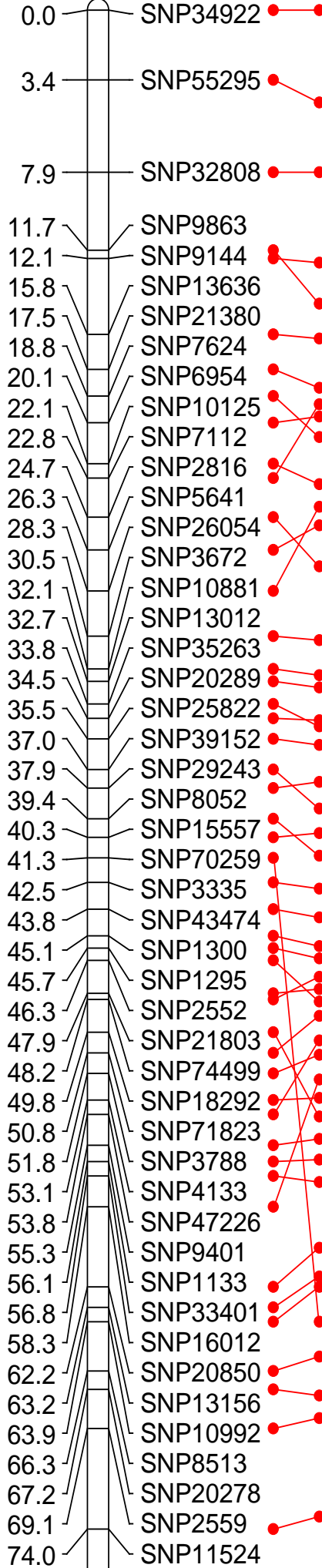

LG17

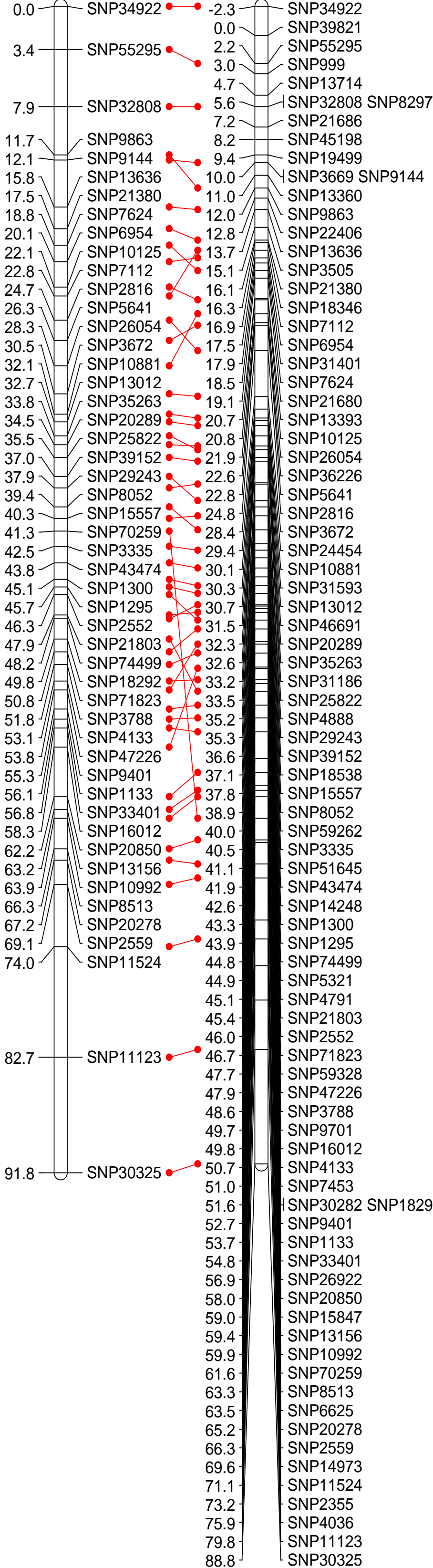

LG17\_M

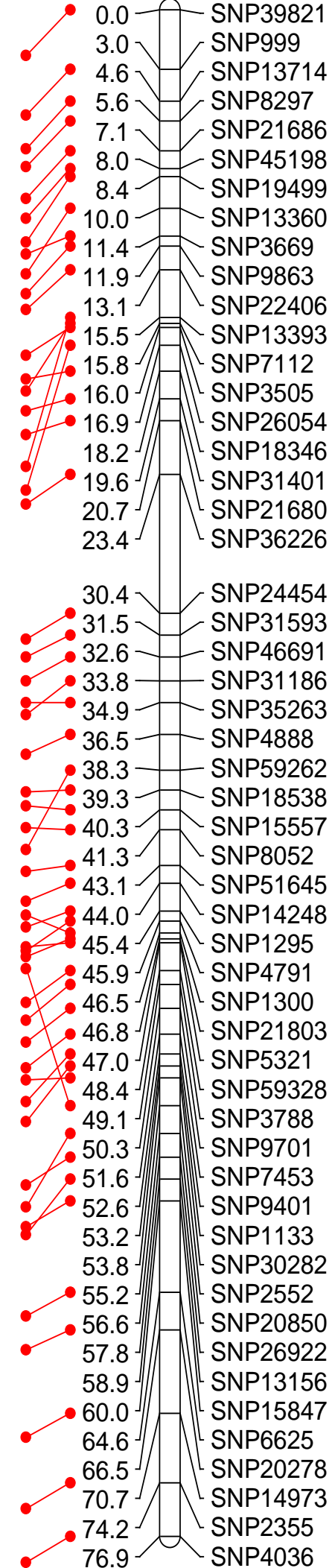

LG16\_F

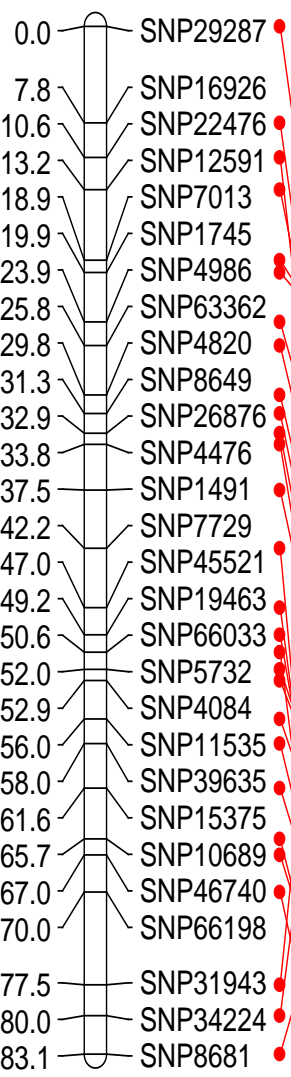

LG16

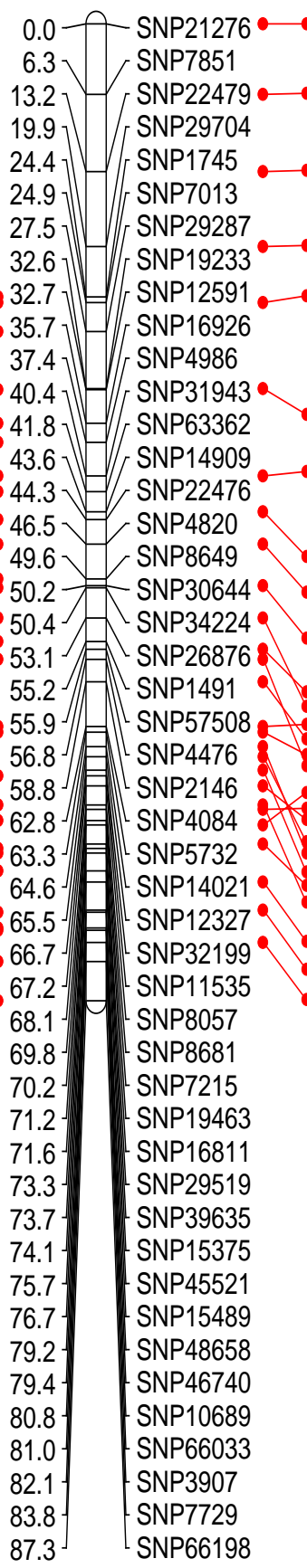

LG16\_M

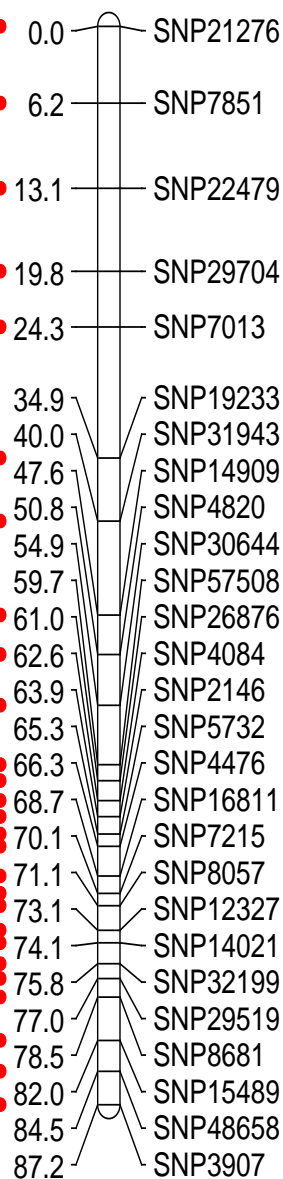

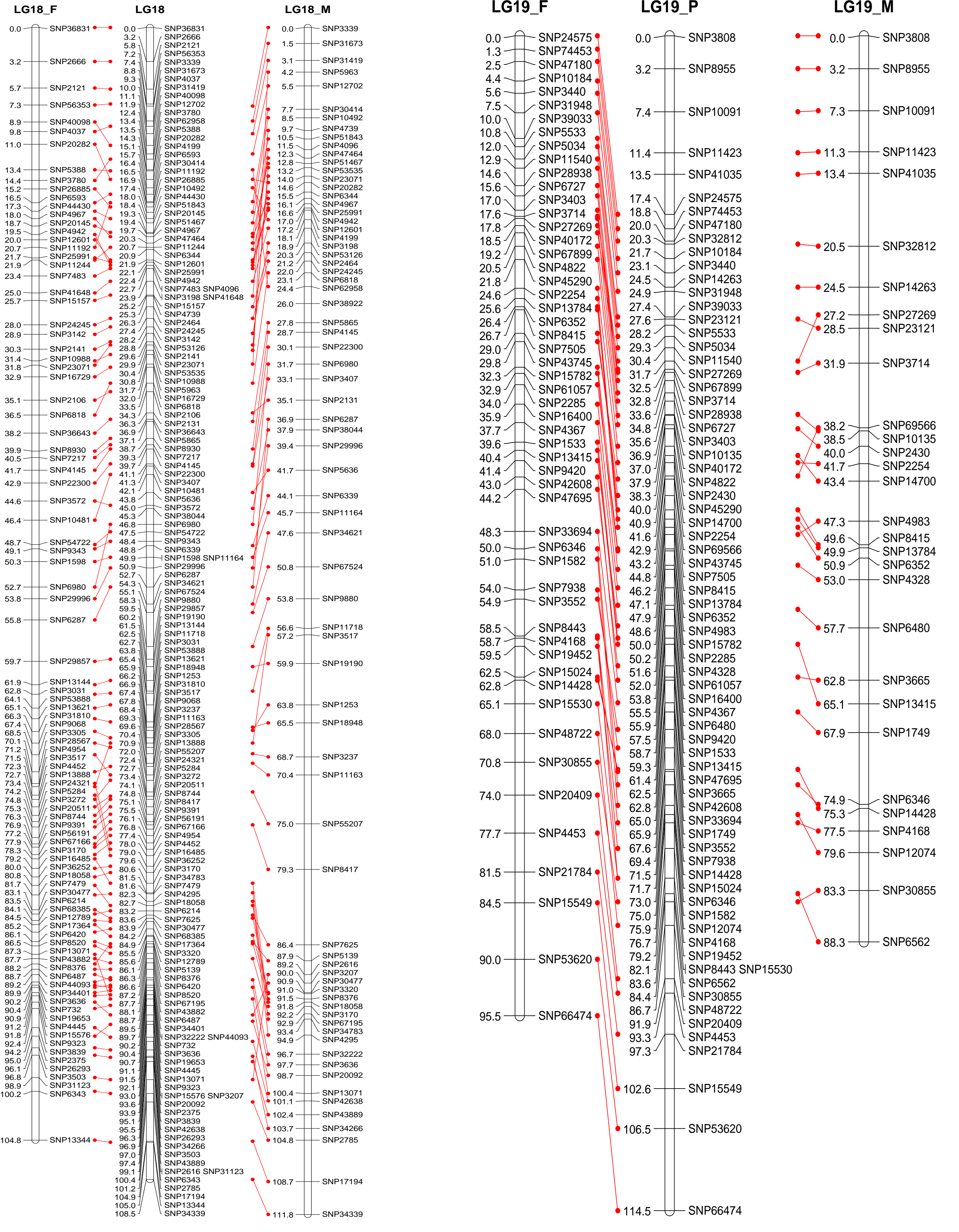

Supplement: Additional files 1 — Table S1. Information on anchor markers. [file 1471-2229-12-148-S1.pdf]

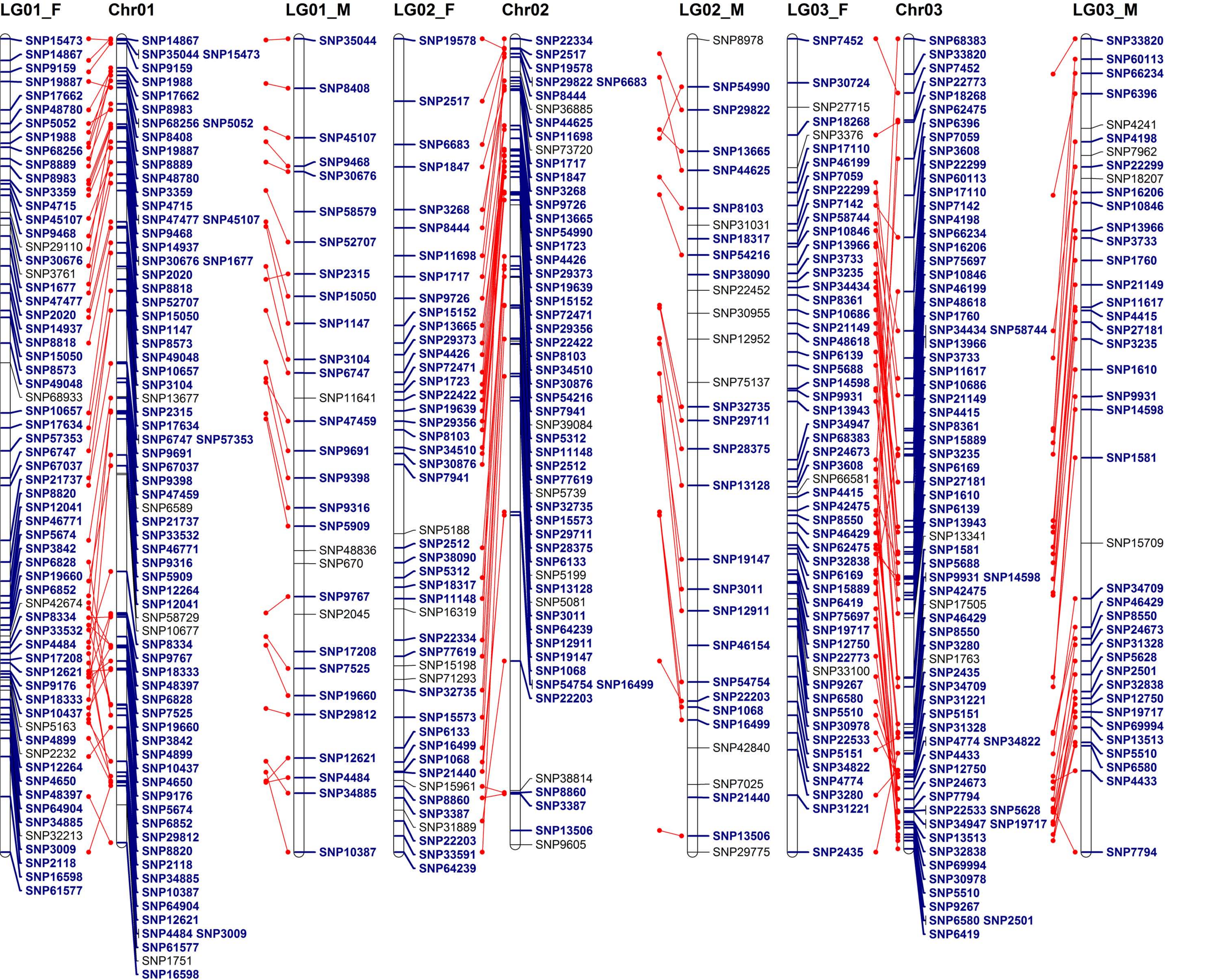

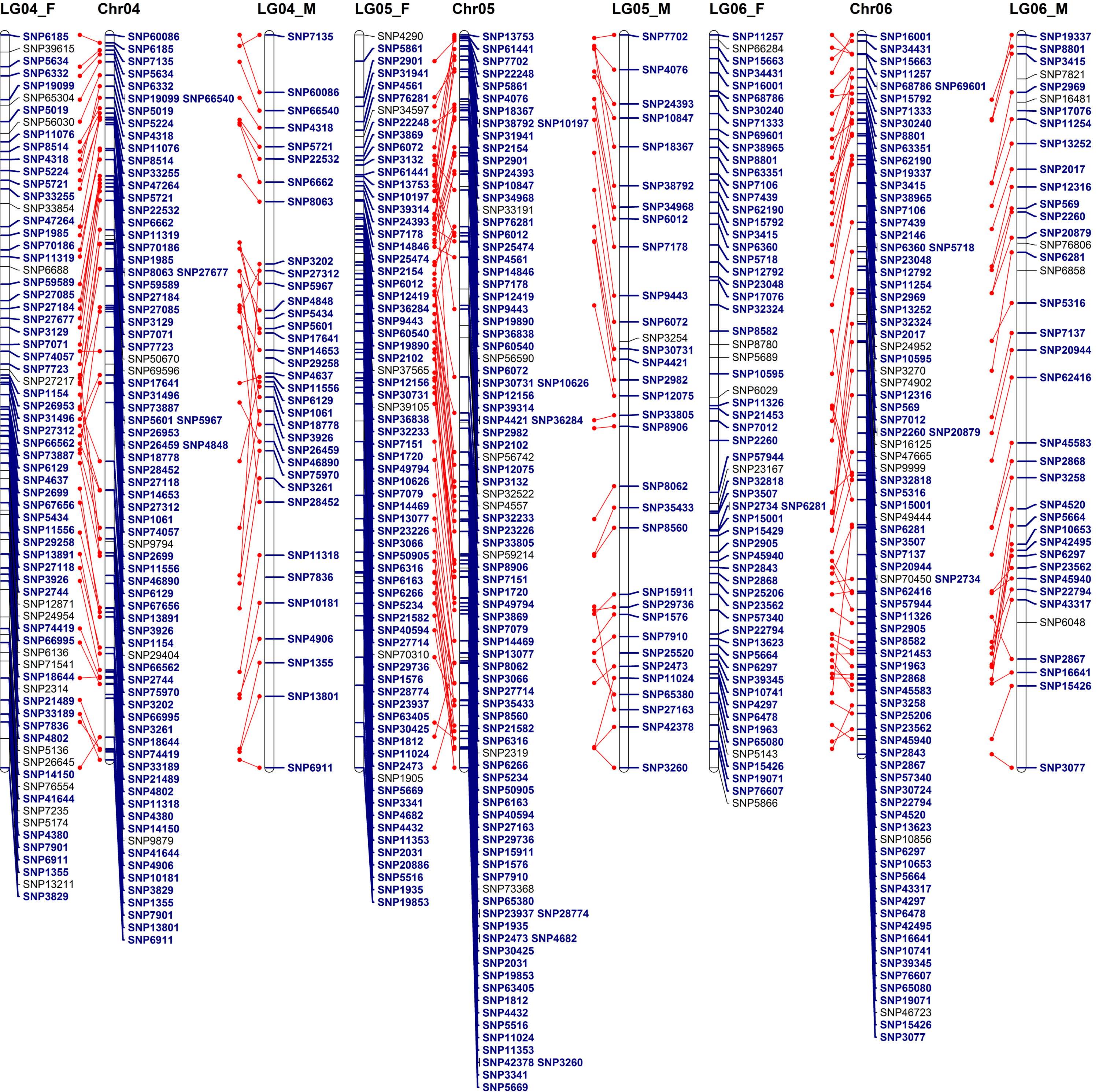

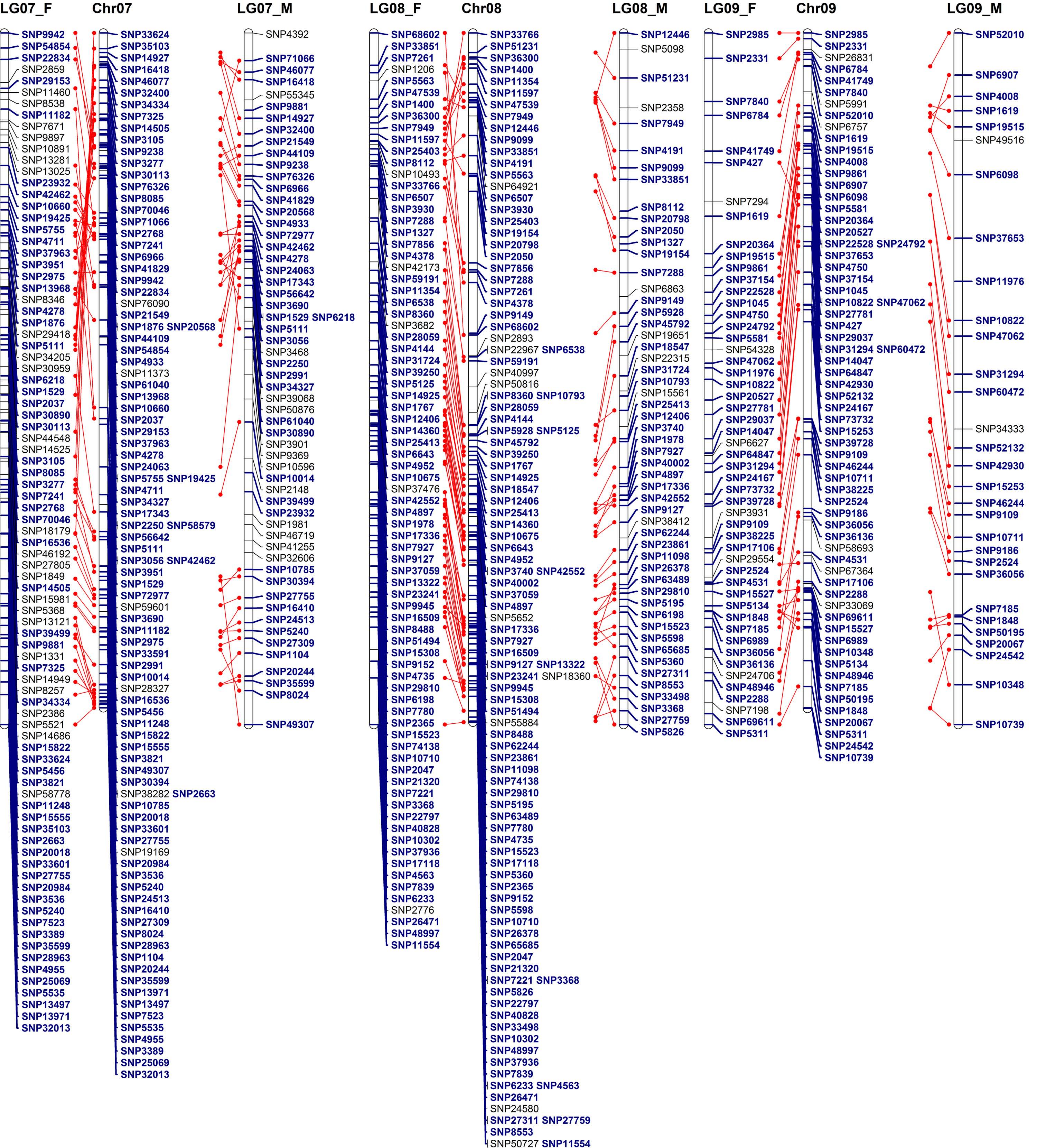

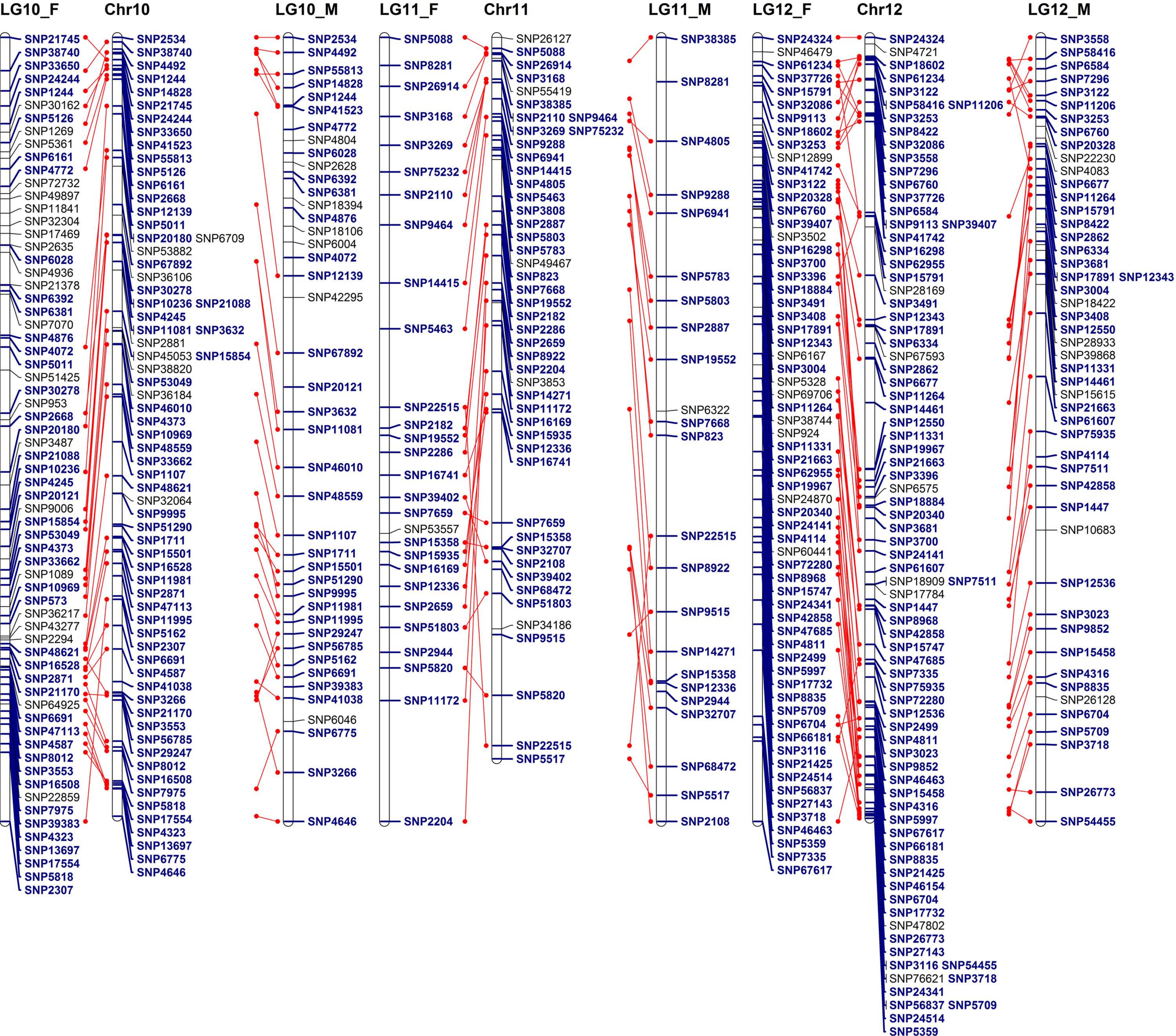

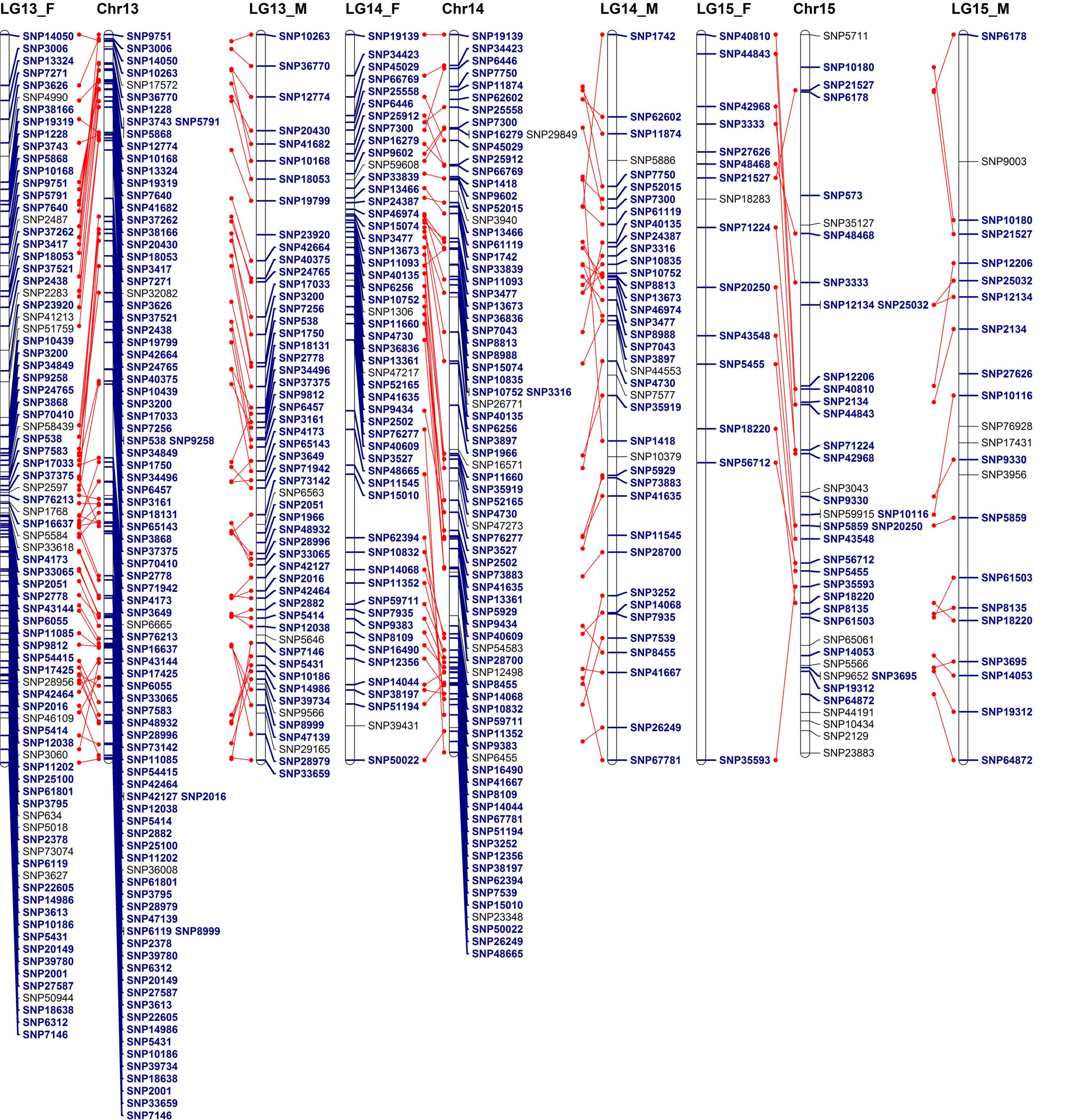

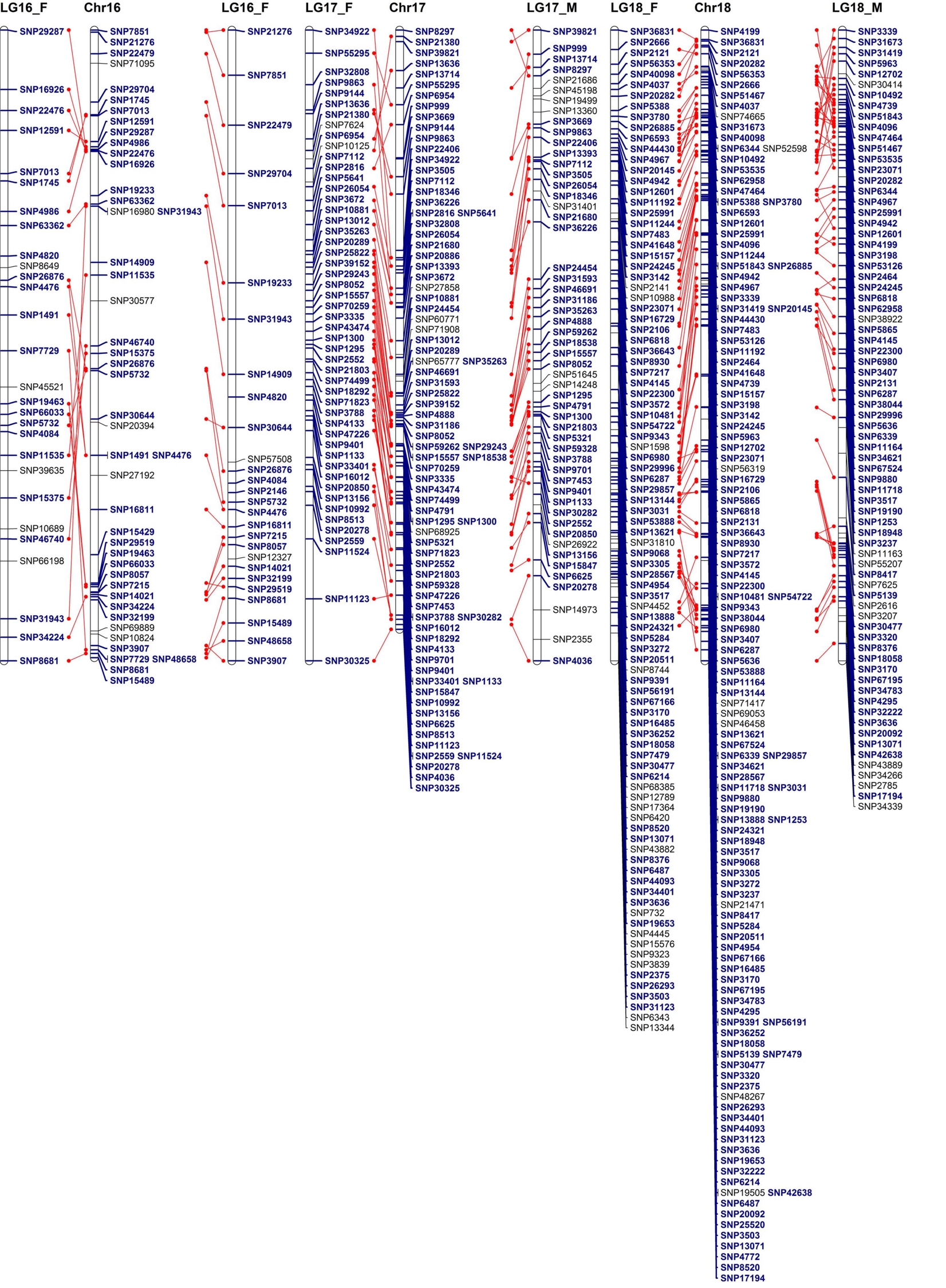

LG19\_F

Chr19

LG19\_M

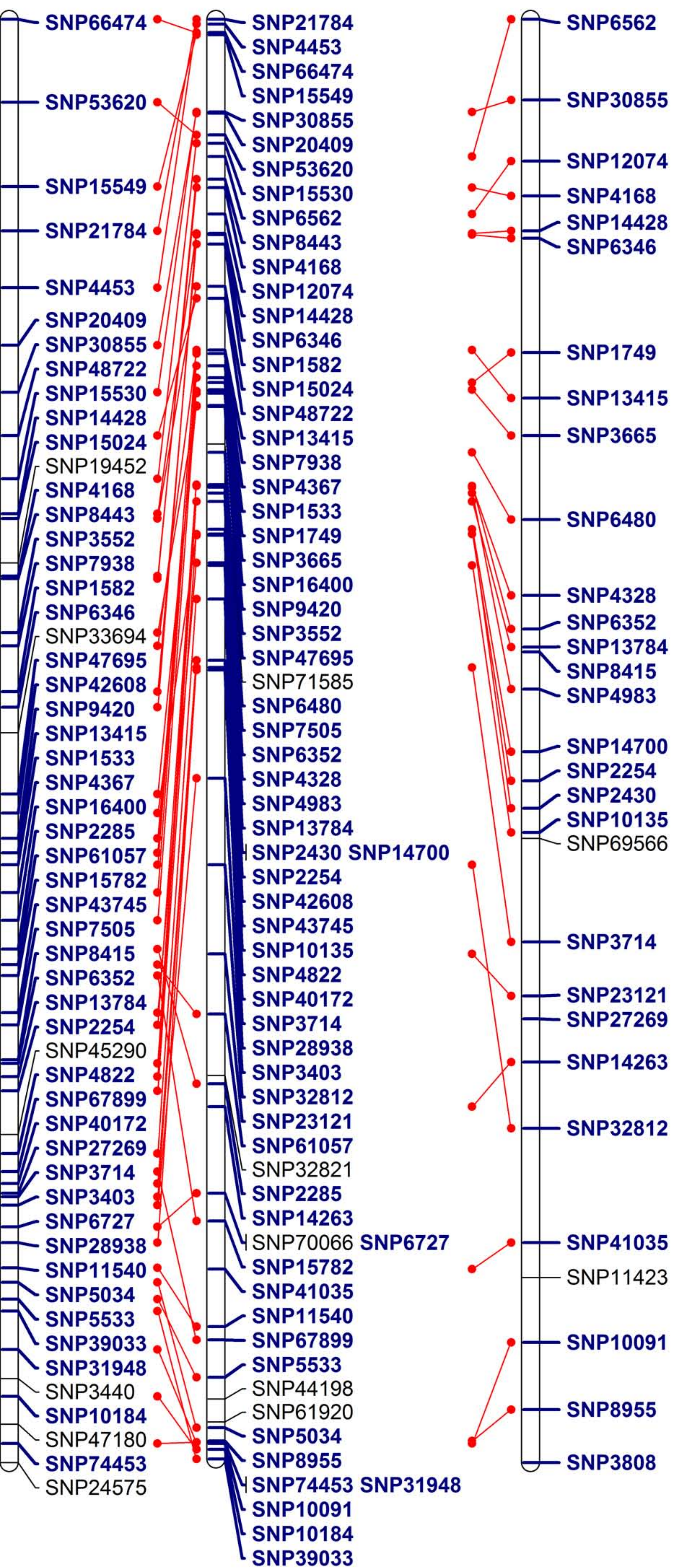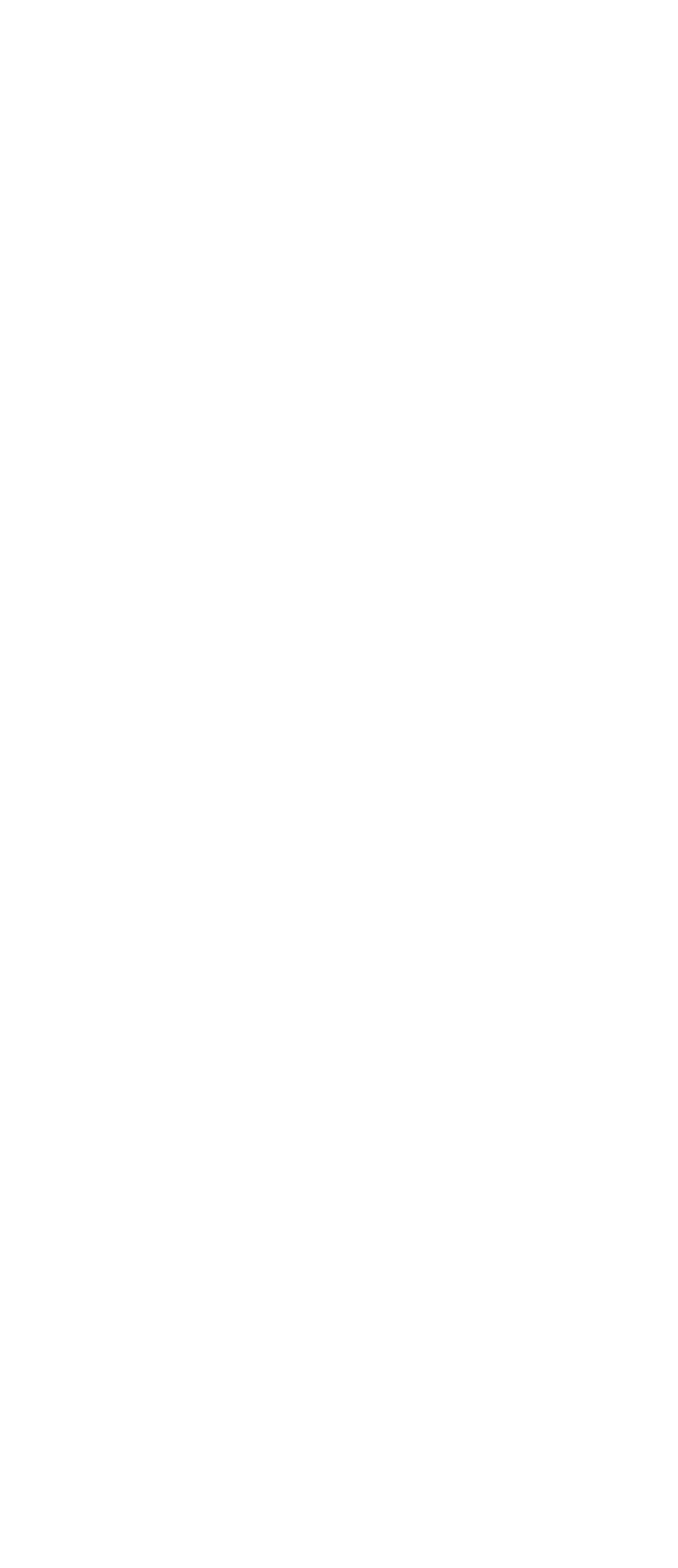

Supplement: Additional files 3 — Table S2. SNP markers on the 19 linkage groups (LGs) and their presented sequence. [file 1471-2229-12-148-S3.pdf]
